# Revisiting the Endophytic Fungi Residing Within *Axonopus compressus*: Molecular Identification, Antagonistic Potential, and Extracellular Enzyme Activities

**DOI:** 10.3390/plants15203229

**Published:** 2026-10-21

**Authors:** Rizwana Kausar, Nurul Farizah Azuddin, Latiffah Zakaria

**Affiliations:** School of Biological Sciences, Universiti Sains Malaysia, Minden, Pulau Pinang 11800 USM, Malaysia; rizwana.kausar111@gmail.com (R.K.); farizah0610@gmail.com (N.F.A.)

**Keywords:** *Axonopus compressus*, fungal endophytes, molecular identification, biological control, hydrolytic enzymes, antifungal activity

## Abstract

Endophytic fungi colonize plant tissues asymptomatically and may contribute to host fitness and protection against biotic stresses. This study characterized the endophytic fungal community associated with healthy leaves of lawn grass (*Axonopus compressus*) and evaluated the extracellular enzyme activities and antagonistic potential of the recovered isolates against selected banana crown rot pathogens. Molecular identification revealed a diverse assemblage of fungal taxa, including species of *Curvularia*, *Colletotrichum*, *Diaporthe*, *Xylaria*, *Fusarium*, *Penicillium*, and *Aspergillus*. In dual-culture assay, all tested isolates exhibited antagonistic activity against the target pathogens, with mycelial growth inhibition ranging from 16.67% to 70.00%. The isolates displayed variable extracellular enzyme profiles, producing cellulase, protease, lipase, pectinase, and amylase at different levels. These differences in enzyme activities suggest functional differentiation among the endophytic fungi and may reflect distinct resource-use strategies within the leaf microenvironment. The observed antagonistic activity may be associated, at least in part, with extracellular enzyme production, although the contribution of individual enzymes to pathogen suppression requires further investigation. Overall, the findings highlight *A. compressus* as a reservoir of functionally diverse, enzyme-producing fungal endophytes with antagonistic potential against banana crown rot pathogens, providing promising candidates for further evaluation as biological control agents for sustainable banana disease management.

## 1. Introduction

Endophytic fungi live within plant tissues, inter- and intracellularly, without causing any visible disease symptoms [1]. These endophytic fungi also promote plant growth and protect the host plant from pathogens by accommodating environmental stress, triggering defense mechanisms, and enhancing productivity and resilience. This mutually beneficial relationship supports both fungi and plants, with the plant offering shelter and nutrients, while endophytes contribute to plant growth and protection [2,3,4].

*Axonopus compressus*, commonly known as lawn grass, is a perennial, stoloniferous species that thrives across humid tropical and subtropical landscapes [5]. Despite its prevalence as a ground cover, the complex endophytic fungal communities inhabiting its tissues remains relatively understudied. Although fungal endophytes are prevalent within tropical grasses [6,7,8,9], the fungal community remains an untapped reservoir for bioactive discovery and as biocontrol agent. Their efficacy for extracellular production and as a biocontrol agent stems from their ability to internally colonize host tissues, allowing them to bypass external environmental fluctuations and withstand diverse biotic and abiotic stresses. These symbionts bolster host fitness through several mechanisms, which are growth promotion, stress resilience, and pathogen antagonism. Endophytic fungi can synthesize a broad range of bioactive compounds, including polysaccharides, lipids, essential minerals, vitamins, enzymes, and phytohormones, which actively stimulate plant development [10,11]. This symbiotic association enhances nutrient acquisition and optimizes the host’s physiological response to environmental stressors [11]. Through pathogen antagonism, which includes niche competition, antibiosis, and mycoparasitism, alongside the induction of host systemic resistance and the production of secondary metabolites, fungal endophytes inhibited pathogen growth and development [12,13].

In the present study, we expanded the sampling areas to reassess the endophytic fungi associated with the leaves of *A. compressus*. Many endophytes occur at low abundance or exhibit sporadic colonization patterns and therefore require greater sampling effort to be reliably detected [11]. Increased sampling effort increases the probability of detecting rare or previously overlooked taxa. Therefore, a more comprehensive sampling approach allowed us to identify a broader range of endophytic fungal taxa. The expanded information on the occurrence of endophytic fungi in the leaves of *A. compressus* provides a foundation for subsequent investigations of their pathogenic potential, host range, and ecological functions.

One main area requiring biocontrol strategies is the management of banana crown rot, a serious postharvest disease responsible for substantial economic losses. Infection typically occurs when opportunistic fungal pathogens colonize the exposed crown tissue during the dehanding process [14]. The disease is particularly challenging as it is associated with a complex of fungal pathogens rather than a single causal agent. Currently, disease management relies on the application of synthetic fungicides. However, the emergence of fungicide-resistant pathogen strains and increasing consumer demand for fungicide-free produce necessitate the development of sustainable, eco-friendly alternatives.

The ecological success of endophytic fungi is driven by their functional roles and biochemical capabilities. To successfully colonize host tissues and acquire nutrients without eliciting a host immune response, these endophytes secrete various extracellular enzymes, including cellulases, proteases, lipases, pectinases, and amylases [15,16]. Characterizing these enzymatic profiles is essential for understanding plant–microbe interactions, as it elucidates the endophytes’ specific functional roles and strategies for biochemical niche partitioning within the host plant [16]. Furthermore, these enzymes not only play a role in nutrient acquisition and asymptomatic colonization but also defense against invading plant pathogens [17].

We hypothesized that *A. compressus* harbors diverse culturable endophytic fungi, some of which exhibit antagonistic activity against banana crown rot pathogens and produce extracellular enzymes that may contribute to their antagonistic potential. Therefore, this study aimed to reassess the culturable endophytic fungi associated with *A. compressus* through expanded sampling across multiple locations, evaluate their in vitro antagonistic activity against banana crown rot pathogens, and determine their capacity to produce extracellular enzymes. The findings are expected to broaden current knowledge of the culturable endophytic fungi associated with *A. compressus* and identify isolates with potential applications in biological control. Furthermore, the assessment of extracellular enzyme production may provide preliminary insights into functional traits associated with these endophytes and their potential ecological roles.

## 2. Results

### 2.1. Identification of Endophytic Fungi

A total of 79 endophytic fungal isolates were successfully isolated from leaves of *A. compressus.* The fungal isolates were confirmed as endophytes as no growth of mycelia was observed in imprinted PDA plates after 7 days of incubation. The surface sterilization method successfully removed epiphytic fungi from the surface of the leaves.

Based on the colony appearance and microscopic observation, the endophytic fungal isolates were grouped to six genera, namely *Curvularia* (*n* = 21), *Colletotrichum* (*n* = 14), *Fusarium* (*n* = 2), *Penicillium* (*n* = 2), *Aspergillus* (*n* = 1), and *Xylaria* (*n* = 1). Due to the limitations of morphological characteristics for genus-level identification, the other endophytic fungal isolates were categorized into eight distinct morphotypes (1–8). The morphological characteristics of the endophytic fungi isolated from leaves of *A. compressus* are shown in Supplementary Figure S1.

Endophytic fungal species identified based on molecular markers and phylogenetic analysis are shown in Table 1. Phylogenetic analysis confirmed the identity of the endophytic fungal isolates which were clustered in the same clade with their epitype strains. The distribution of endophytic fungal species across the six sampling locations is presented in Supplementary Table S1.

Endophytic fungal isolates of *Curvularia* and *Colletotrichum* were identified based on ITS and GAPDH sequences. Based on GAPDH sequences, *Curvularia* isolates showed 99% similarity with *C. lunata*, *C. geniculata*, *C. ischaemi*, and *C. akaiiensis* (Table 1; Figure 1 and Figure 2). The Maximum Likelihood (ML) phylogenetic tree showed that isolates of *Curvularia* from the same species were grouped in the same clade with their epitype strains (Figure 1). *C. lunata* isolates formed a well-supported clade with the *C. lunata* reference sequence (CBS 730.96), with 99% bootstrap support. Similarly, isolates of *C. geniculata*, *C. akaiiensis*, and *C. ischaemi* clustered with their respective reference sequences and received 99% bootstrap support. Therefore, isolates of *Curvularia* were identified as *C*. *lunata*, *C. geniculata*, *C. ischaemi*, and *C. akaiiensis* (Table 1, Figure 1).

Five species of *Colletotrichum* were identified as *C. gloeosporioides*, *C. siamense*, *C. grevilleae*, *C. queenslandicum*, and *C. gigasporum.* The ITS and GAPDH sequences of the endophytic *Colletotrichum* isolates showed 99–100% similarity to *C. gloeosporioides*, *C. siamense*, *C. grevilleae*, *C. queenslandicum*, and *C. gigasporum* (Table 1). The grouping of the *Colletotrichum* isolates in the ML tree showed that isolates from the same species were grouped in the same clade with their reference strains (Figure 2). In the ML tree, isolates LK22, LK20, LK12, CN15, and EH33 clustered with the *C. siamense* reference strain MFLU090230, with 99% bootstrap support. Isolates EH20 and LK21 clustered with the *C. queenslandicum* reference strain ICMP 1778, with 100% bootstrap support. Isolates LK16, CN4, Li4, and LK11 formed a well-supported clade with the *C. gloeosporioides* reference strain ICMP 17821, with 100% bootstrap support. Isolates Li16 and CN1 clustered with the *C. grevilleae* reference strain CBS 132879, with 100% bootstrap support. Similarly, isolate EH26 clustered with the *C. gigasporum* reference strain CBS 133266, with 100% bootstrap support.

Basic Local Alignment Search Tool (NCBI web BLAST) analysis of ITS and ACT sequences of *Xylaria* EH22 showed 99% similarity to *Xylaria feejeensis* (Table 1). In the ML tree, isolate *Xylaria* EH22 clustered with the epitype strain of *X. feejeensis* (565HSTJF), forming a strongly supported clade with 100% bootstrap support, indicating its identity as *X. feejeensis* (Figure 3).

BLAST analysis of TEF-α and β-tubulin sequences of Morphotype 1 isolates showed 99% similarity to *Diaporthe shawiae* (Table 1). Maximum Likelihood tree further demonstrated that *D. shawiae* isolates clustered with the epitype strain of *D. shawiae* (BRIP 64534a) in clade A (Figure 4) forming a strongly supported clade with 100% bootstrap support. Therefore, Morphotype 1 isolates were phylogenetically identified as *D. shawiae*.

BLAST analysis of ITS sequences showed that isolates of Morphotypes 2–8 exhibited 99% similarity to *Stagonospora bicolor* (Morphotype 2), *Bipolaris oryzae* (Morphotype 3), *Muyocopron laterale* (Morphotype 4), *Achroiostachys humicola* (Morphotype 5), *Beltraniella endiandrae* (Morphotype 6), *Dimorphiseta obtusa* (Morphotype 7), and *Xenocylindrocladium subverticillatum* (Morphotype 8) (Table 1). The species identities of these morphotypes were further supported by ML phylogenetic analyses, which demonstrated that isolates representing each morphotype clustered with their corresponding reference strains (Figure 5, Figure 6 and Figure 7).

In the ML tree, isolates LK10, LA9, LA21, and IH16 clustered with the *B. oryzae* reference strain CBS 127242, with 76% bootstrap support (Figure 5a). Together with the 99% sequence similarity obtained from BLAST analysis, this phylogenetic relationship provided additional support for the identification of the isolates as *B. oryzae*, although the bootstrap value indicated moderate support for the grouping. Isolates LK8, LK6, Li2, EH34, EH7, EH12, EH31, CN16, CN9, CN19, Li12, Li10, and Li3 clustered with the *S. bicolor* reference strain ATCC 42652, with 99% bootstrap support (Figure 5b). In Figure 6a, isolates EH27 and EH35 clustered with the *B. endiandrae* reference strain CBS 137976, with 100% bootstrap support, whereas isolates EH25, EH8, EH16, and Li17 clustered with the *M. laterale* reference strain CBS 145313, with 99% bootstrap support (Figure 6b).

In the ML tree, isolates EH11 and EH36 both clustered with the *X. subverticillatum* reference strain CBS 113660, with 100% bootstrap support (Figure 7a). Similarly, isolates IH20 and EH28 clustered with the *D. obtusa* reference sequence MH793290, also with 100% bootstrap support (Figure 7b). Isolates LA27 and LA17 clustered with the *A. humicola* reference strain CBS 868.73, with 99% bootstrap support (Figure 7c).

Endophytic *Penicillium citrinum*, *Aspergillus aculeatus*, and *Fusarium equiseti* were identified based on ITS sequence and ML phylogenetic analysis. BLAST results showed 99% similarity of the ITS sequences to *P. citrinum*, *A. aculeatus*, and *F. equiseti* (Table 1). This was further supported by an ML phylogenetic tree, where the isolates clustered with their corresponding epitype strains (Figure 8), confirming their identities. In the ML tree, isolate LA11 clustered with the *A. aculeatus* reference strain CBS 172.66, with 100% bootstrap support (Figure 8a). Isolates LA1 and LA6 clustered with the *P. citrinum* reference strain CBS 139.45, with 100% bootstrap support (Figure 8b). Similarly, isolates CN13 and LK18 clustered with the *F. equiseti* reference strain CBS 307.94, with 100% bootstrap support (Figure 8c).

### 2.2. In Vitro Antagonistic Activity

Dual-culture test showed that all tested fungal endophytes from the leaves of *A. compressus* demonstrated antagonistic activity against mycelial growth of banana crown rot pathogens (Table 2). The percentage of inhibition varies from 16.67 to 70.00%, with minimal to moderate interactions. The type of interactions between the fungal endophytes and the pathogens also varies. The most common interactions were type E, with mutual inhibition occurring at a greater distance (>2 mm), and type D, with mutual inhibition occurring at a short distance (<2 mm). The highest inhibition was shown by *D. shawiae* LA15 against *F. verticillioides* with PGI of 70.00% and *C. siamense* (PGI 66.16%) (Figure 9A,B) with type B interaction, of which the endophyte overgrows the pathogen (Table 2). Four fungal endophytes, *C. geniculata* EH18, *C. gloeosporioides* LK11, *F. equiseti* CN13, and *D. shawiae* LA15, inhibited the mycelial growth of all tested banana crown rot pathogens, with percentages of inhibition from 50.56 to 70.00%, displaying moderate interaction. The other fungal endophytes produced minimal to low interactions, with percentages of inhibition from 16.67 to 50.00%. The results highlight a range of antagonistic activity of endophytic fungal isolates from the leaves of *A. compressus*.

### 2.3. Extracellular Enzymes Activity

Screening of extracellular enzyme production among the endophytic fungal isolates revealed variable abilities for cellulase, protease, lipase, pectinase, and amylase activities (Table 3). All the tested fungal endophytes produced at least two of the five enzymes analyzed. *Xylaria feejeensis* was the only species capable of producing all five extracellular enzymes, exhibiting moderate activity across each enzyme and thus indicating broad enzymatic potential. Protease activity was detected in all isolates, with comparatively higher activity observed in *C. lunata*, *C. geniculata*, *B. oryzae*, and *M. laterale*. The highest cellulase activity was recorded in *X. feejeensis*, whereas *P. citrinum* and *X. subverticillatum* showed no detectable cellulase activity. Lipase activity was highest in *C. gloeosporioides*, *C. siamense*, and *F. equiseti*. *Muyocopron laterale* exhibited the highest pectinase activity, while the highest amylase activity was observed in *C. ischaemi* (Table 3).

## 3. Discussion

Identification of endophytic fungi using the ITS region offers several key advantages. As the primary DNA barcode for fungi, its high sequence variability makes it ideal for distinguishing fungal species [18]. Additionally, the ITS region is widely utilized in phylogenetic and systematic studies [19] because it can be readily amplified across diverse fungal taxa using universal primers [20]. Furthermore, extensive publicly accessible databases containing ITS sequences greatly facilitate accurate species identification [21].

However, the ITS region may not effectively differentiate species within a species complex or a closely related species, like *Colletotrichum*, *Curvularia*, and *Diaporthe*. This limitation could stem from reduced sequence variation among many closely related species and the presence of sequence heterogeneity among ITS copies [22]. Consequently, multiple markers were utilized to accurately identify the endophytic fungal isolates and performed phylogenetic analysis. The choice of genes varies depending on the fungal genera of which genes such as TEF-1α, β-tubulin, GAPDH, and ACT were employed in the present study.

Although the ITS region is widely used as a primary molecular marker for fungal identification, it may provide insufficient resolution for distinguishing closely related species within certain genera, including *Fusarium*, *Penicillium*, and *Aspergillus*. In the present study, isolates identified as *F. equiseti*, *P. citrinum*, and *A. aculeatus* showed 99% ITS sequence similarity to their respective reference strains. However, morphological characterization was limited to the genus level, and the ITS sequence similarity alone may not provide definitive species-level identification. This limitation is particularly relevant to *F. equiseti*, as closely related species within the *Fusarium incarnatum-equiseti* species complex may not be reliably distinguished using ITS sequences alone. Although ITS has been recommended for the initial identification of *Aspergillus* species, additional markers, such as TEF-1α and RNA Polymerase II Second Largest Subunit (RPB2) for *Fusarium*, β-tubulin (BenA) and calmodulin (CaM) for *Penicillium*, and CaM and BenA for *Aspergillus*, would provide greater resolution for species-level identification [23,24,25]. Therefore, the identification of these three species based on ITS sequences is regarded as provisional, and further multilocus sequence analyses are recommended to confirm their taxonomic identities.

Several fungal genera identified in this study such as *Curvularia*, *Colletotrichum*, *Fusarium*, *Xylaria*, *Diaporthe*, *Aspergillus*, and *Penicillium* are well-documented as generalists with broad host ranges. The recovery of fungal genera such as *Curvularia*, *Colletotrichum*, *Fusarium*, *Xylaria*, *Diaporthe*, *Aspergillus*, and *Penicillium* suggested a Class 3 non-clavicipitaceous endophytic strategy, characterized by horizontal transmission, high spatial dispersal, and broad host range [26,27]. Many of these generalist taxa are known to synthesize diverse secondary metabolites with antimicrobial, antioxidant, and insecticidal properties, which may facilitate survival across varied host environments and potentially contribute to host defense [28]. Additionally, the high isolation recovery of these generalist taxa from *A. compressus* leaves likely stems from their rapid growth rates. As noted by Arnold and Lutzoni [27], generalist species belonging to *Xylaria*, *Colletotrichum*, *Diaporthe*, and *Fusarium* grow rapidly and outcompete slower endophytes on nonselective plant-based media commonly used for isolation, such as malt extract, potato dextrose, and cornmeal agar.

This study relied on culture-dependent methods, and the retrieval of endophytes was inherently limited to culturable isolates. In culture-dependent approaches, fast-growing fungi tend to dominate the culture medium and restrict the physical space and nutrients available for slower-growing species, thereby suppressing their development and outcompeting them [29,30]. Additionally, factors like nutrition, temperature, aeration, tissue size, and the quantity of plant samples can influence the number and growth of the isolated fungal endophytes [31].

Endophytic *C. grevilleae*, *C. ischaemi*, *B. endiandrae*, *B. oryzae*, and *X. subverticillatum* have not been documented as endophytes in any plant. While *D. shawiae* was recovered from *Chloris gayana*, a Poaceae [32], the findings did not clarify if the species was an endophyte, pathogen, or saprophyte. The present study provides the second record of *D. shawiae* found in Poaceae. *Dimorphiseta obtusa* has been isolated from the roots of turfgrass [33], but it was not specified whether it is an endophyte.

Several fungal endophytes isolated from the leaves of *A. compressus* have been identified as endophytes in Poaceae. The endophytic *C. lunata* has been recovered in aromatic tall grass (*Cymbopogon caesius*) [34], barnyard grass (*Echinochloa glabrescens*) [35], lemon grass (*Cymbopogon citratus*) [36], and *A. compressus* [9]. The presence of endophytic *S. bicolor* in lawn grass was also documented by Azuddin et al. [9]. Endophytic *P. citrinum* has been isolated from three grass species, *Digitaria bicornis*, *Alloteropsis cimicina*, and *Heteropogon contortus* [37,38]. As for endophytic *A. humicola*, this endophyte was recovered in *Phragmites australis* [39], as well as in a rice variety found in Sri Lanka [40].

Among the fungal endophytes identified, *X. feejeensis*, *F. equiseti*, *C. siamense*, and *P. citrinum* have been reported as biocontrol agents against plant pathogens [41,42,43,44], whereas certain isolates of *C. gloeosporioides*, *C. siamense*, and *C. lunata* have been reported as promising biocontrol of weeds [45,46,47]. The results suggested that several species of fungal endophytes from leaves of *A. compressus* have the potential to act as biocontrol agents of plant pathogens and weeds. To our knowledge, the literature regarding the antagonistic activity of endophytic *C. geniculata*, *C. akaiiensis*, *C. grevilleae*, *C. gigasporum*, *B. oryzae*, *S. bicolor*, *M. laterale*, *B. endiandrae*, *D. obtusa*, *X. subverticillatum*, and *D. shawiae* against plant pathogens remains sparse. Consequently, this study offers initial evidence documenting the antagonistic potential of these endophytic fungal taxa.

The present study identified a broader range of fungal endophyte species than Azuddin et al. [9], likely due to our expanded sampling site and larger leaf sample size used for isolation. A broader range of fungal endophyte species is essential in the symbiotic relationship between the endophytes and their host plant as different fungal species contribute to enhancing plant health, improving stress tolerance, and defending against pathogens. As Li et al. [48] noted, a diverse endophytic fungal assemblage can enhance host resilience by conferring functional versatility under fluctuating environmental conditions.

The antagonistic activity of fungal endophytes recovered from leaves of *A. compressus* against four banana crown rot pathogens in dual-culture tests may be attributed to several mechanisms, including competition for nutrients and space, secretion of extracellular hydrolytic enzymes, and production of bioactive secondary metabolites. Competition for nutrients and space may restrict pathogen growth by limiting the resources and space available for mycelial development. In addition, the production of extracellular hydrolytic enzymes may contribute to antagonistic interactions through the degradation of susceptible structural components of fungal pathogens. Some fungal endophytes may also produce volatile and non-volatile secondary metabolites with antifungal properties that inhibit pathogen growth [49]. However, the specific mechanisms responsible for the observed antagonistic activity were not directly investigated in the present study. Therefore, these mechanisms remain hypothetical and require further experimental validation.

Further studies are required to verify the biocontrol potential of the fungal endophytes and assess their practical applications. Subsequent investigations should include greenhouse and field trials to evaluate the effectiveness of the fungal endophytes in suppressing plant diseases under different environmental conditions. These trials should also assess the ability of the fungal endophytes to colonize host plant tissues without causing disease symptoms, thereby providing further evidence of their suitability as potential biocontrol agents [50].

Extracellular enzyme production has been proposed as one of the mechanisms by which endophytic fungi may suppress pathogen growth [51]. The biocontrol potential of fungal antagonists may be associated with their ability to produce extracellular enzymes, including cellulases, proteases, lipases, pectinases, and amylases, which perform various functions in substrate degradation, nutrient acquisition, and microbial interactions [52]. Certain extracellular enzymes, particularly those targeting fungal structural components, may contribute to pathogen inhibition through disruption of hyphal integrity and cellular structures [53].

In the present study, the endophytic isolates exhibited varying abilities to produce cellulase, protease, lipase, pectinase, and amylase. These enzymatic activities may contribute to nutrient acquisition and interactions with other microorganisms, including fungal pathogens [54]. Cellulases and pectinases are primarily involved in the degradation of plant structural polysaccharides [55], whereas proteases may act on proteins associated with fungal structures [56]. Lipases may contribute to lipid degradation and nutrient utilization [57]. However, the present study did not directly establish a relationship between extracellular enzyme production and pathogen inhibition. Therefore, the potential contribution of individual enzymes to the observed antagonistic activity remains hypothetical.

The antagonistic activity observed in the dual-culture tests demonstrated the in vitro inhibitory potential of the endophytic fungi but did not establish their biocontrol efficacy under plant or field conditions. Further investigations, including enzyme activity–antagonism correlation analyses and mechanistic studies, would be necessary to determine whether specific extracellular enzymes contribute to pathogen suppression.

Endophytic fungi have been reported to produce lipases, which may contribute to their ability to interact with and colonize host plants [58]. Together with proteases, lipases may facilitate fungal penetration through plant tissues by modifying structural components of the host [59].

In the present study, protease activity was detected in all tested isolates, with *C. lunata*, *C. geniculata*, *B. oryzae*, and *M. laterale* exhibiting relatively high activity. Lindstrom et al. [60] suggested that proteases may contribute to fungal–plant interactions by modifying host structural barriers and facilitating nutrient acquisition. Protease production may also enable fungal endophytes to utilize plant proteins as nutrient sources, although their potential involvement in the degradation of host defense-related proteins requires further investigation [61]. However, the specific role of proteases in host colonization and maintenance of the endophytic relationship was not directly investigated in the present study.

The production of cellulases and pectinases by fungal endophytes has been associated with their ability to utilize plant structural components and may contribute to their adaptation to different host tissues [62]. These extracellular enzymes may have several ecological functions, including nutrient acquisition and interactions with other microorganisms, and have also been proposed to contribute to host defense or latent pathogenicity [17]. In the present study, a high proportion of the tested isolates produced cellulases and pectinases. This enzymatic capability may indicate their potential to utilize different plant substrates and could be consistent with the ability of some endophytic fungi to occupy different ecological niches during their life cycle [63]. However, the present study did not determine whether these enzyme activities are associated with a transition between pathogenic and saprophytic lifestyles.

Starch represents an important energy reserve in plant tissues. As endophytic fungi are already present within host tissues, their amylase activity may enable them to utilize starch as a potential carbon source, particularly when plant tissues undergo senescence or degradation [64,65]. Thus, amylase production may contribute to nutrient acquisition by endophytic fungi and potentially influence their interactions with other microorganisms in the host tissue [15].

In the present study, the tested fungal endophytes exhibited different extracellular enzyme activities, which may contribute to their ability to utilize a range of substrates within the leaf environment. For example, the relatively high pectinase activity of *M. laterale* may indicate its potential to utilize pectin-rich substrates, whereas the amylase activity detected in *C. akaiiensis* suggests a potential capacity for starch utilization. Such differences in enzymatic capabilities may contribute to niche differentiation and resource utilization within the leaf mycobiome [27,66]. However, whether these differences result in resource partitioning or reduced competition among endophytic fungi was not directly assessed in the present study and remains to be investigated.

These extracellular enzymatic capabilities may also contribute to the ecological interactions between endophytic fungi and plant pathogens, potentially influencing nutrient competition and pathogen suppression within host tissues. However, although several endophytic isolates exhibited antagonistic activity against banana crown rot pathogens in vitro, the relationship between their extracellular enzyme production, host colonization, and biocontrol potential remains to be established.

The culture-dependent method used in this study is considered when interpreting the composition of endophytic fungi recovered from *A. compressus*. Culture-dependent methods favor the recovery of fungi capable of growing under the specific nutritional and incubation conditions employed. Consequently, rapidly growing and readily culturable fungi may be preferentially recovered, whereas slow-growing or difficult-to-culture taxa may be overlooked or outcompeted by rapidly growing colonies [67,68,69]. The fungal taxa recovered therefore represents those detectable under the conditions employed in this study and should not be interpreted as reflecting their actual relative abundance within the plant or the complete endophytic mycobiome. Differences in surface sterilization, tissue size, culture medium, and incubation period can further influence the number and identity of fungal endophytes recovered [70]. Thus, some members of the *A. compressus* endophytic fungal community, particularly taxa with specialized nutritional requirements or limited culturability, may not have been represented among the isolates obtained.

This limitation is particularly relevant when comparing the present findings with studies based on culture-independent approaches. Environmental sequencing and DNA metabarcoding can detect fungal taxa that are difficult to recover under conventional laboratory conditions and may therefore reveal greater species richness or differences in fungal community composition compared with culture-dependent isolation [71]. However, these approaches are complementary rather than interchangeable. Culture-independent methods enable broader detection of fungal communities, whereas culture-dependent methods provide living isolates that can be morphologically characterized, preserved in culture collections, and experimentally evaluated for biological activities [71]. Therefore, the fungal taxa recovered in the present study represents the culturable endophytic fungal assemblage associated with *A. compressus* under the isolation conditions employed. Future studies integrating culture-dependent isolation with culture-independent metabarcoding could provide a more comprehensive understanding of the endophytic fungal diversity associated with *A. compressus*, including taxa that are difficult to recover or grow slowly under conventional culture conditions. Despite this limitation, the culture-dependent approach was appropriate for the functional objectives of the present study, as it enabled the recovered isolates to be evaluated for antagonistic activity and extracellular enzyme production.

## 4. Materials and Methods

### 4.1. Sampling Location

Lawn grass samples were collected between March 2020 and December 2021 from the Universiti Sains Malaysia (USM) main campus, Penang Island, Malaysia. Leaves of the lawn grass were collected from six locations within the USM campus where the grass was planted: Harapan Lake; Hamzah Sendut Library; International House; USM Convocation Expo; Vector Control Research Unit; and Eco Hub, USM. Healthy leaves were randomly collected to increase the likelihood of recovering both commonly and less frequently isolated endophytic fungal species. A total of 180 leaves (30 leaves per location) were collected from the six sampling locations. Penang Island has a warm tropical climate throughout the year, with daily temperatures generally ranging from 24 °C to 33 °C and rainfall generally increasing from September towards the wetter months at the end of the year. Therefore, the sampling period encompassed both relatively drier and wetter conditions.

The leaves were put in paper envelopes and brought to Plant Pathology Lab, School of Biological Sciences, USM, for isolation process. In the lab, the leaves were placed in a beaker and rinsed under running tap water for 2 min to wash any debris or dirt on the surface of the leaves.

### 4.2. Isolation of Endophytic Fungi

Surface sterilization was used to isolate endophytic fungi from lawn grass leaves. The leaves were soaked in 70% ethanol for 2 min, rinsed in sterile distilled water for 30 s, and soaked in 5% sodium hypochlorite for 2 min. The leaves were then rinsed three times in sterile distilled water for 1 min each and gently patted dry with sterile filter paper. The sterilized leaves were cut into small pieces (about ~5 mm) using a sterile scalpel and placed on Potato Dextrose Agar (PDA) (HiMedia Laboratories, Dombivli (Thane district) Maharashtra, India). Four leaf segments (approximately 5 mm each) were placed on each PDA plate. The plates were incubated at room temperature (25 ± 1 °C) until growth of mycelia was observed from the leaf tissues. The mycelia were then transferred to new PDA plates.

To determine the effectiveness of surface sterilization, the imprint method was applied [10]. The effectiveness of surface sterilization was assessed using the imprint method. All surface-sterilized leaf fragments were pressed onto fresh PDA plates and incubated for 7 days. No fungal growth was observed on the imprint plates, indicating the absence of detectable culturable surface-associated fungi under the conditions tested. Fungi subsequently recovered from the surface-sterilized leaf tissues were therefore considered endophytic fungi. The recovered isolates represent the culturable endophytic fungi obtained from the sampled *A. compressus* leaves during the study period.

### 4.3. Molecular Identification of Fungal Isolates

Prior to molecular identification, the endophytic fungal isolates were tentatively grouped into their respective genera based on colony morphology and microscopic features, including conidial shape, conidiophore structure, conidial head arrangement, and septation. Isolates that cannot be identified to the genus level based on morphological characteristics are categorized as morphotypes. The references used for morphological identification are presented in Supplementary Table S2.

For DNA extraction, the mycelia were grown in Potato Dextrose Broth (PDB). The fungal cultures were incubated at 25 ± 1 °C for 2–7 days. *Penicillium* and *Aspergillus* isolates were incubated for 2 days to prevent over-sporulation, while all other genera were incubated for 7 days. After incubation period, the mycelia grown on the surface of PDB were harvested and dried on sterilized filter paper. The dried mycelia were then ground with liquid nitrogen to a fine powder using sterile mortar and pestle. The mycelial powder (0.8–0.10 g) was transferred to a 1.5 mL centrifuge tube and placed in a freezer before DNA extraction. Genomic DNA of the endophytic fungi was extracted using a DNA extraction kit (Invisorb^®^ Spin Mini Kit, Berlin, Germany) according to the manufacturer’s instructions.

The molecular markers used for species identification were selected based on published studies and their established suitability for resolving species within the respective fungal genera. These markers included the internal transcribed spacer (ITS) region, glyceraldehyde-3-phosphate dehydrogenase (GAPDH), β-tubulin, actin (ACT), and translation elongation factor 1-alpha (TEF-1α). The selected markers, primer sequences, and supporting references are provided in Supplementary Tables S3 and S4.

FOR PCR amplification, a PCR reaction mixture was prepared in a total volume of 50 µL containing 8 µL of 5X Green GoTaq^®^ Flexi Buffer; 8 µL of 25 mM MgCl_2_; 1 µL of 10 mM dNTP mix (Promega, Madison, WI, USA); 8 µL each of 5.0 µM for the ITS region, TEF-1α, β-tubulin, GAPDH, and ACT primers; 0.3 µL of 5U/µM GoTaq^®^ DNA polymerase (Promega, Madison, WI, USA); and 0.6 µL of the DNA template. The reaction mixture was made up to a final volume of 50 µL using sterile distilled water.

PCR amplification was carried out in a thermal cycler, MycyclerTM PCR (Bio-Rad Laboratories, Hercules, CA, USA). PCR cycles for amplification of the ITS region and β-tubulin: initial denaturation at 94 °C for 85 s, 35 cycles of denaturation at 95 °C for 35 s, annealing at 55 °C, extension at 72 °C for 90 s, and a final extension at 72 °C for 10 min; GAPDH: initial denaturation at 95 °C for 4 min, 35 cycles of denaturation at 95 °C for 30 s, annealing at 60 °C for 30 s, extension at 72 °C for 7 min, and a final extension at 72 °C for 10 min; ACT: initial denaturation at 94 °C for 5 min, 41 cycles of denaturation at 95 °C for 30 s, annealing at 61 °C for 45 s, extension at 72 °C for 90 s, and a final extension at 72 °C for 90 s; TEF-1α: initial denaturation at 95 °C for 5 min, 35 cycles of denaturation at 95 °C for 30 s, annealing at 60 °C for 45 s, extension at 72 °C for 90 s, and a final extension at 72 °C for 7 min.

Agarose gel (1%) was employed to visualize PCR products in 1X Tris-borate-EDTA (TBE) buffer, which was stained using FloroSafe DNA stain (Axil Scientific, Singapore). The PCR products were sent to a service provider for DNA sequencing (NHK Bioscience Solutions, Kuala Lumpur, Malaysia).

### 4.4. Phylogenetic Analysis

Following sequencing, a consensus sequence was generated by aligning the forward and reverse DNA sequences through pairwise alignments with ClustalW using Molecular Evolutionary Genetics Analysis software version 11 (MEGA11) [72]. The consensus sequences were modified as required, and a BLAST search was conducted against the GenBank database. The sequences were compared with reference sequences available in the National Center for Biotechnology Information (NCBI) database using the Basic Local Alignment Search Tool (BLASTN) through the NCBI Web BLAST interface (BLAST: Basic Local Alignment Search Tool: https://blast.ncbi.nlm.nih.gov/Blast.cgi, accessed on between 20 June 2022 and 15 January 2023.

Each isolate was identified based on the highest sequence similarity match from a BLAST search against the GenBank database. A similarity threshold of ≥97% was applied for species-level identification, whereas values between 95% and 97% were used for genus-level identification [73]. To confirm identity, phylogenetic analyses were performed using relevant genetic markers and reference sequences of closely related taxa from GenBank.

Phylogenetic trees were generated from the combined sequences derived from multiple sequence alignments using MEGA11 software. A Maximum Likelihood (ML) tree was generated with 1000 bootstrap replicates. We employed a heuristic ML approach known as the nearest neighbor interchange (NNI), where an initial tree for ML is automatically produced. The optimal model for the ML tree was identified through a model search that utilized five discrete gamma categories. The model test applied to generate the ML tree is shown in Supplementary Table S5. Reference strains were chosen from the epitype or type strains of each genus, as well as from the closest matches from the BLAST search results (Supplementary Table S6).

### 4.5. In Vitro Antagonistic Activity

Sixteen isolates of endophytic fungi isolated from the leaves of *A. compressus*, namely *C. akaiiensis* LA5, *C. lunata* IH3, *C. geniculata* EH18, *C. gloeosporioides* LK11, *C. siamense* CN15, *F. equiseti* CN13, *X. feejeensis* EH22, *P. citrinum* LA1, *D. shawiae* LA15, *S. bicolor* EH34, *B. oryzae* LA21, *M. laterale* EH16, *A. humicola* LA27, *B. endiandrae* EH27, *D. obtusa* IH20, and *X. subverticillatum* EH11, were selected to evaluate their antagonistic activity against pathogens of banana crown rot. The isolates represent a wide range of genera that increases the likelihood of identifying isolates with varied mechanisms of antagonism.

The banana crown rot pathogens were isolated from Berangan banana (*Musa acuminata* AAA genome group) collected from fruit stalls and markets in several locations in Peninsular Malaysia [74]. The pathogens were *Fusarium proliferatum* KL77, *Fusarium verticilliodes* NG52, *Neocosmospora ipomoeae* PH32, and *Colletotrichum siamense* KL89, identified based on morphological and molecular characteristics and confirmed as causal agents of banana crown rot through pathogenicity tests [74].

The antagonistic activity of the fungal endophytes against banana crown rot pathogens was evaluated using a dual-culture assay. A 5 mm diameter mycelial plug was cut from the actively growing margin of a 3-day-old culture of each endophytic fungus and pathogen and placed on opposite sides of a 9 cm diameter Petri plate containing PDA, with the centers of the two plugs positioned approximately 6 cm apart. For each pathogen–endophyte combination, three replicate plates were prepared. Control plates consisted of a 5 mm diameter mycelial plug of the respective pathogen cultured alone on PDA. The plates were incubated at room temperature for eight days. The experiment was conducted twice independently.

After eight days of incubation, the radial growth of the pathogen was measured in the direction of the endophytic fungus. The percentage of growth inhibition (PGI) was calculated according to Korsten and De Jager [75]:

(1) $$\mathrm{PGI}\left(\%\right)=\frac{R1-R2}{R1}\times 100$$

R1: measured from the inoculation point to the edge of the pathogen colony on the control plate. R2: measured from the inoculation point to the edge of the colony on the dual-culture plate in the direction of the endophytes.

Based on the calculated PGI values, growth inhibition categories (GICs) ranging from 0 to 4 were assigned, following the scale by Skidmore and Dickinson [76]. A GIC of 0 indicated an absence of antagonistic activity or no inhibition; 1—minimal inhibition (1–25% growth inhibition); 2—low antagonistic (26–50% growth inhibition); 3—moderate inhibition (51–75% growth inhibition); and 4—high inhibition (76–100% growth inhibition). The PGI values were analyzed separately for each pathogen using one-way analysis of variance (ANOVA), followed by Tukey’s HSD test for comparison of means at *p* < 0.05 by employing SPSS software version 25.

The interactions between endophytic fungi and plant pathogens were classified into five types (A–E) according to the criteria described by Skidmore and Dickinson [76]. Interaction types were determined by visually examining the colony growth patterns and the presence or absence of inhibition zones in the dual-culture tests. Type A was characterized by intermingling growth of the pathogen and endophytic fungus, whereas type B indicated overgrowth of the pathogen by the endophytic fungus, and type C indicated overgrowth of the endophytic fungus by the pathogen. Types D and E represented mutual inhibition, characterized by clear inhibition zones of less than 2 mm and greater than 2 mm, respectively, between the opposing colonies.

### 4.6. Extracellular Enzymes Activity

We tested the same 16 isolates used in the antagonistic activity for their ability to produce various extracellular enzymes, including cellulase, protease, lipase, pectinase, and amylase. The extracellular enzyme production was measured by the appearance of a clear zone or halo surrounding the colony, and the enzyme activity index (EAI) was calculated after the incubation period. After 5–10 days of incubation, the colony of the fungal endophytes and the formation of a halo (clear zone) or precipitating zone around the colony were measured and recorded, and EAI was calculated. In some enzymes, the halo appeared after adding some reagent to the medium. Enzyme activity index (EAI) was calculated based on the following formula by [15].

(2) $$\mathrm{Enzyme activity index}\;(\mathrm{EAI})=\frac{\mathrm{Diameter of halo zone}\;(\mathrm{cm})}{\mathrm{Diameter of fungal growth}\;(\mathrm{cm})}$$

Based on EAI values calculated, the enzymatic reaction of the isolates was classified according to the reaction category (Table 4) described by [15].

Cellulase production was assessed by culturing isolates on PDA supplemented with 0.5% carboxymethylcellulose. Following a 5-day incubation at 26 ± 1 °C, plates were stained with 0.2% Congo Red and decolorized with 1 M NaCl. Cellulase activity was confirmed by the presence of a distinct yellow clearance zone [77].

Protease activity was screened by culturing fungal plugs on Nutrient Agar (NA) (HiMedia^®^, Maharashtra, India) supplemented with 8% gelatin as a substrate, following the method of Hankin and Anagnostakis [78]. After a 7-day incubation at 26 ± 1 °C, proteolytic activity was visualized by overlaying plates with a saturated ammonium sulfate solution. This treatment formed a precipitate that highlighted distinct opaque clearance zones around positive colonies.

To detect lipase, fungal isolates were cultured on a modified peptone agar (10 g/L peptone, 5 g/L NaCl, and 0.1 g/L CaCl_2_·2H_2_O) supplemented with 1% Tween 20. After 7 days of incubation at 26 ± 1 °C, the presence of a precipitation halo around the colony indicating the formation of calcium salt was recorded as an indication of lipase activity [78].

Endophytic fungal isolates were screened for pectinase production on citrus pectin medium (10 g/L) supplemented with mineral salts [78]. After 5 days of incubation at 26 ± 1 °C, 1% hexadecyltrimethylammonium bromide was applied to the medium. Pectinase activity was confirmed by the presence of clear zones against a white background, formed by the precipitation of undegraded pectin by the reagent.

Amylase production was evaluated by culturing 5 mm mycelial plugs on NA (HiMedia, Maharashtra, India) supplemented with 0.2% (*w*/*v*) soluble starch at pH 6.0. Following a 7-day incubation at 26 ± 1 °C, the plates were treated with an iodine solution (% iodine in 2% potassium iodide) for 30–60 s. The presence of a clear halo against a dark blue background indicating the hydrolysis of starch confirmed positive amylolytic activity [78].

## 5. Conclusions

This study successfully isolated and identified various species of fungal endophytes from *A. compressus* leaves. These fungal endophytes showed antagonistic activity against banana crown rot pathogens, *F. proliferatum*, *F. verticillioides*, *N. ipomoeae*, and *C. siamense*. To the best of our knowledge, this study provides initial evidence regarding the antagonistic potential of 11 endophytic fungal species including *C. geniculata*, *B. oryzae*, and *D. shawiae* against postharvest pathogens. These findings contribute to the growing literature on fungal biocontrol agents and indicate that *A. compressus* may serve as a promising, underexplored reservoir of antagonistic fungal endophytes with diverse enzymatic profiles. The observed pathogen inhibition suggests that such endophytic fungal isolates might hold potential for integrated disease management applications in tropical fruit production.

Their application appears to align well with ecological and sustainable agricultural practices. However, further studies remain necessary to assess their effectiveness, biosafety, and overall function under field conditions. Continued research into these endophytes may facilitate the development of biocontrol agents, potentially helping to reduce reliance on synthetic fungicides. The observed substrate utilization and extracellular enzyme activities suggest that fungal endophytes within the *A. compressus* mycobiome could possess specialized functional roles. Secretion of diverse extracellular enzymes might aid in modifying plant cell wall components and modulating host defenses to support asymptomatic colonization. Once established, these endophytes may minimize direct competition through biochemical resource partitioning, potentially enabling diverse taxa to coexist within the leaf tissue.

## Figures and Tables

**Figure 1:**
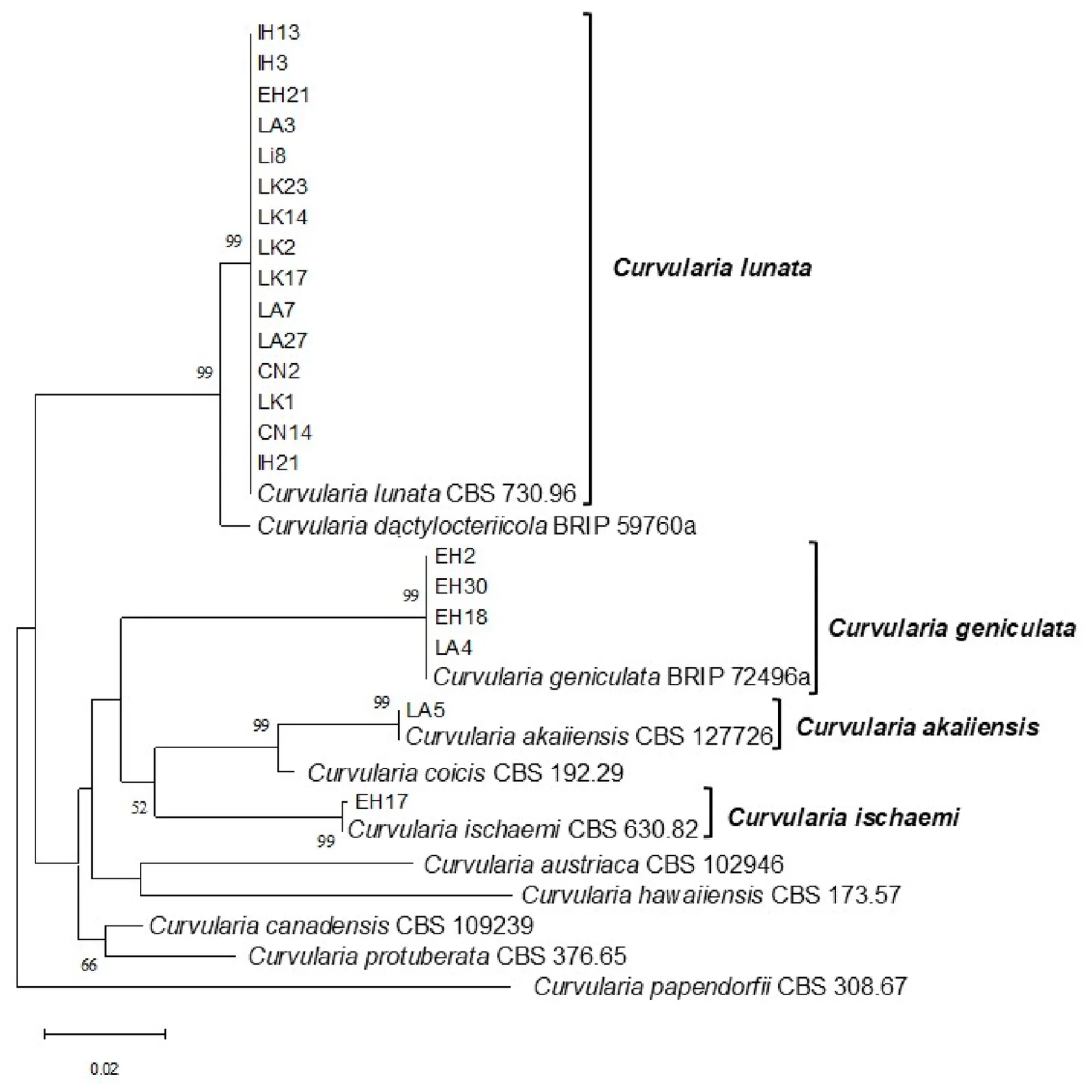
Maximum Likelihood tree inferred from combined sequences of ITS and GAPDH of *Curvularia* spp. isolated from leaves of *A. compressus*. Bootstrap values greater than or equal to 50% are indicated.

**Figure 2:**
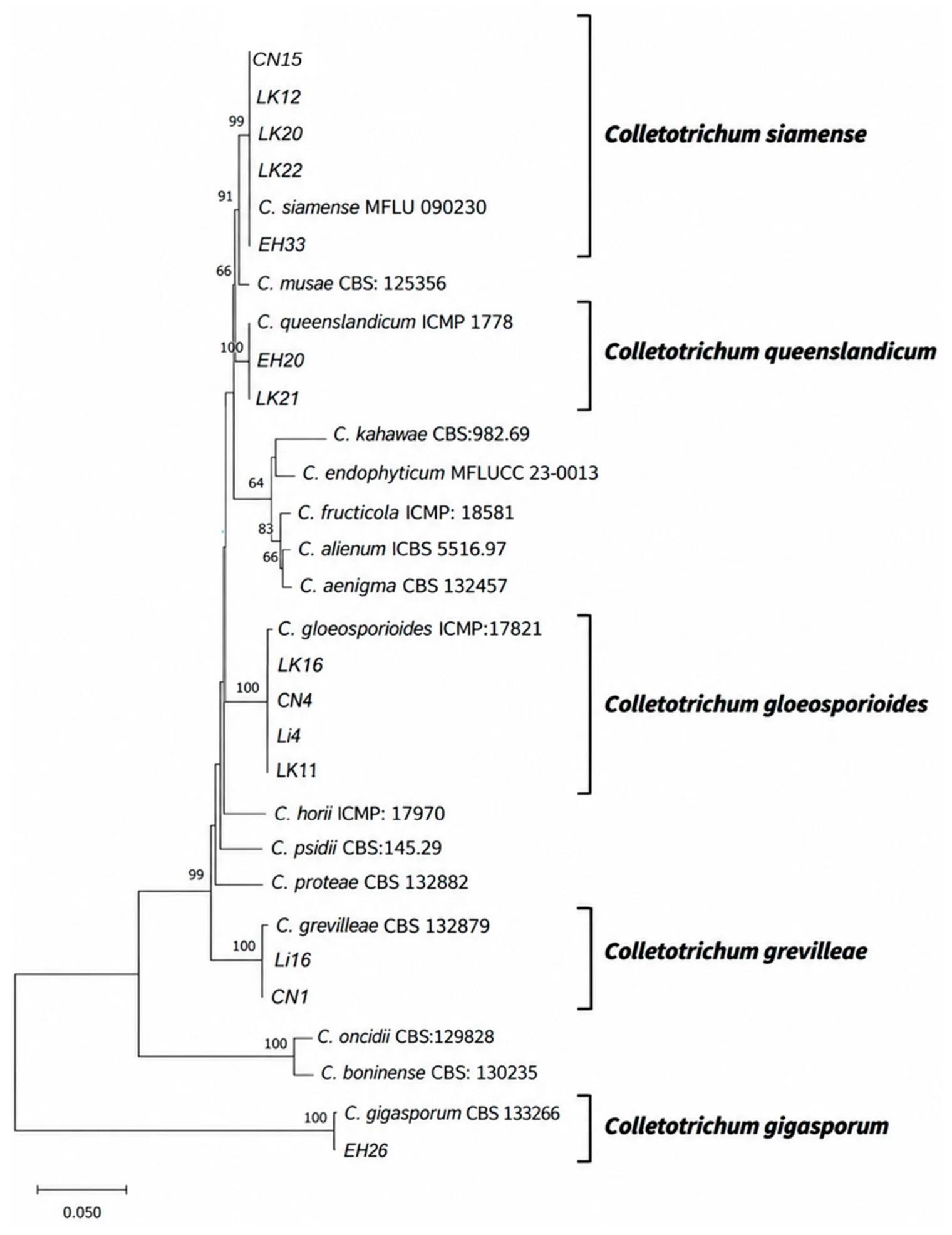
Maximum Likelihood tree inferred from combined sequences of ITS and GAPDH of *Colletotrichum* spp. isolated from leaves of *A. compressus*. Bootstrap values greater than or equal to 50% are indicated.

**Figure 3:**
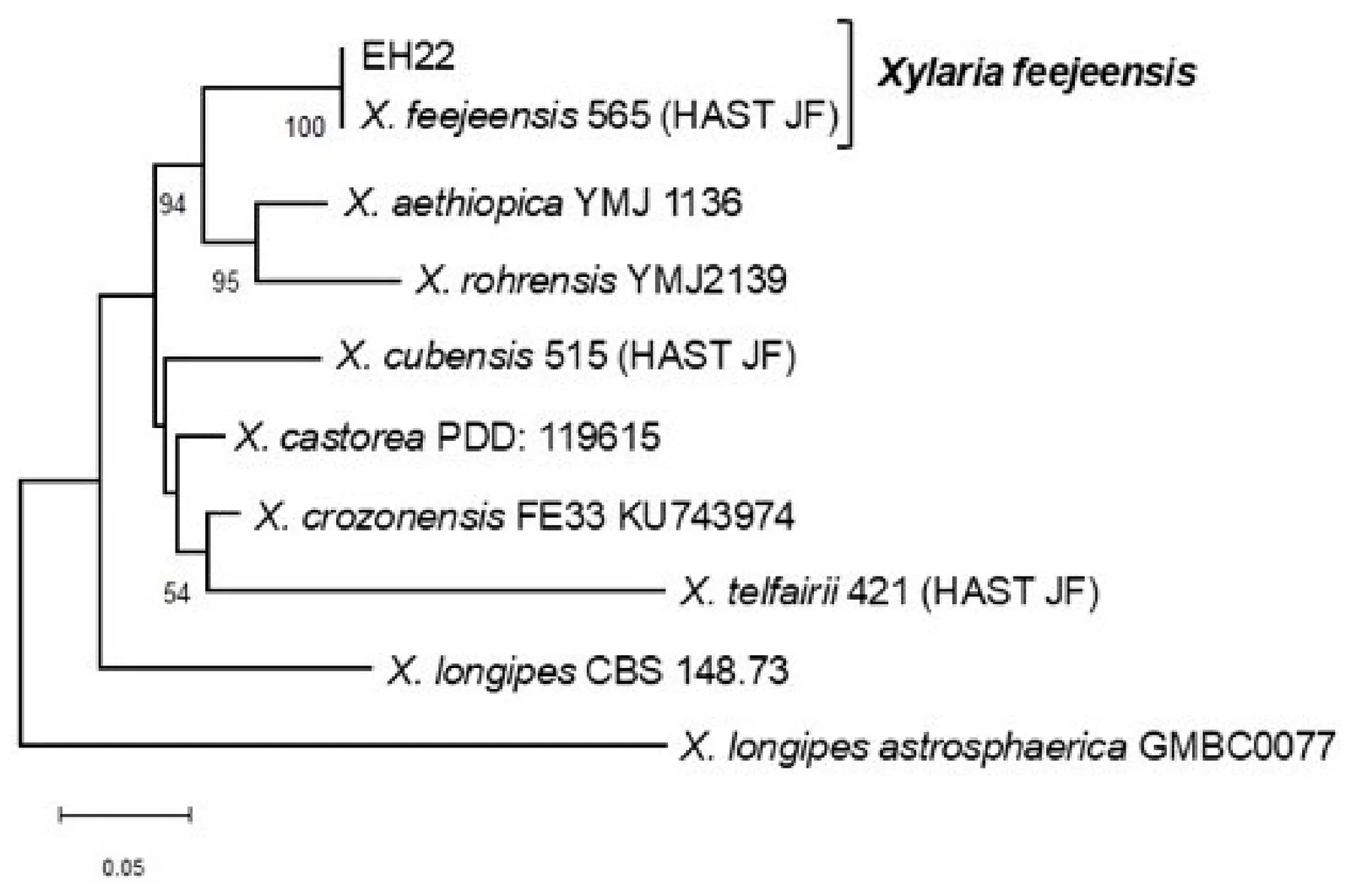
Maximum Likelihood tree inferred from combined ITS and ACT sequences of *Xylaria feejeensis* from leaves of *A. compressus*. Bootstrap values greater than or equal to 50% are indicated.

**Figure 4:**
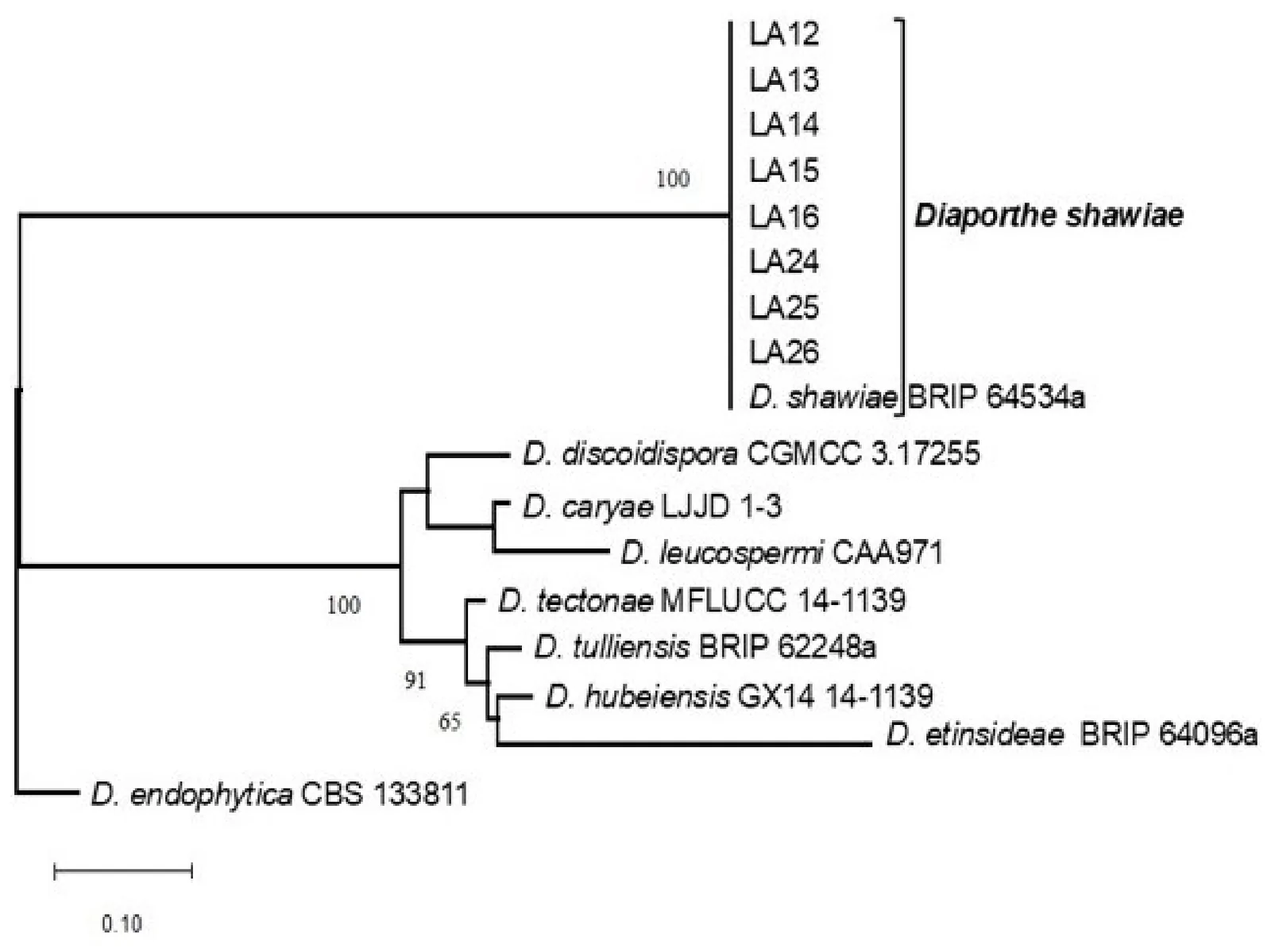
Maximum Likelihood tree inferred from the combined sequences of ITS and β-tubulin of *Diaporthe shawiae* isolated from leaves of *A. compressus*. Bootstrap values greater than or equal to 50% are indicated.

**Figure 5:**
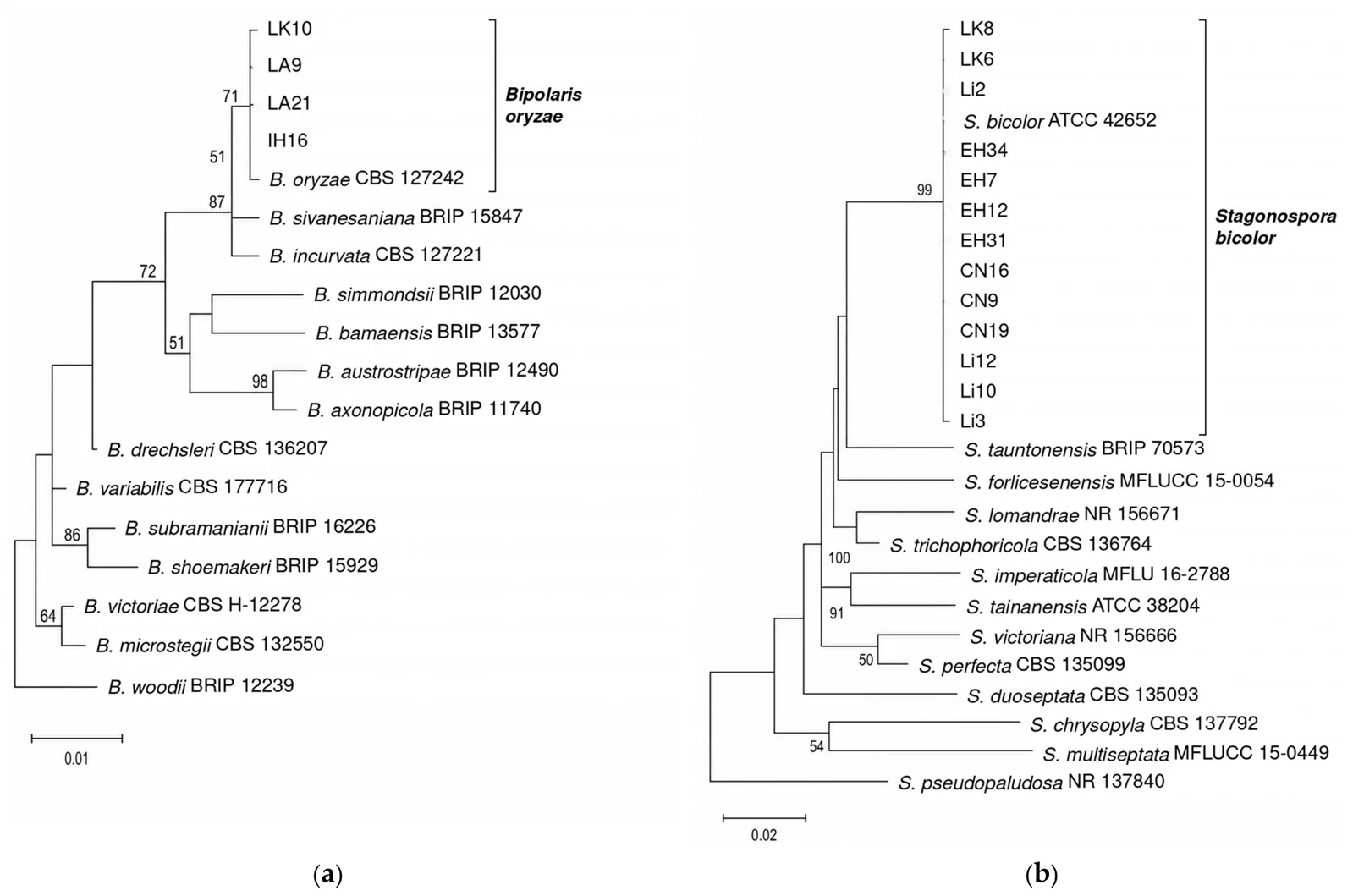
Maximum Likelihood tree inferred from ITS sequences of *Bipolaris oryzae* (**a**) and *Stagonospora bicolor* (**b**) isolated from leaves of *A. compressus*. Bootstrap values greater than or equal to 50% are indicated.

**Figure 6:**
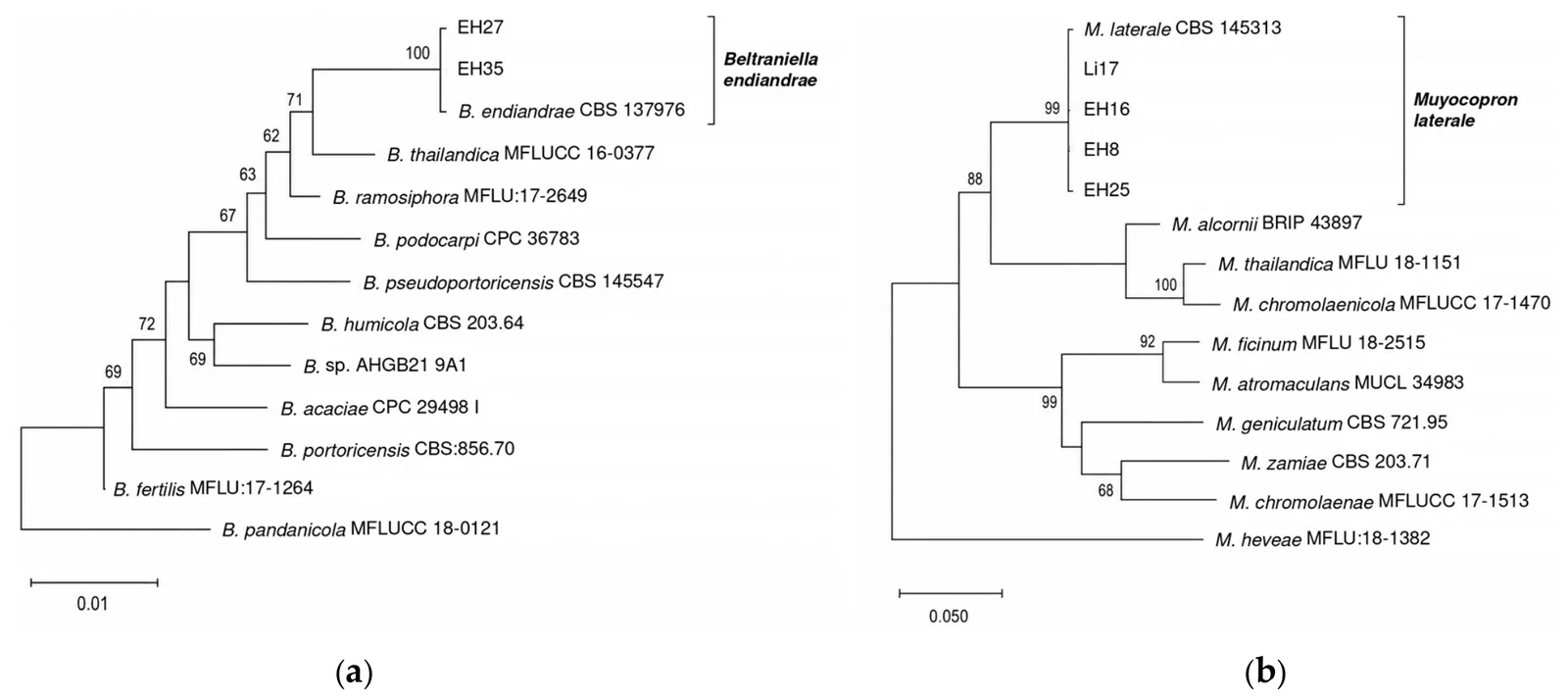
Maximum Likelihood tree inferred from ITS sequences of *Beltraniella endiandrae* (**a**) and *Muyocopron laterale* (**b**) isolated from leaves of *A. compressus*. Bootstrap values greater than or equal to 50% are indicated.

**Figure 7:**
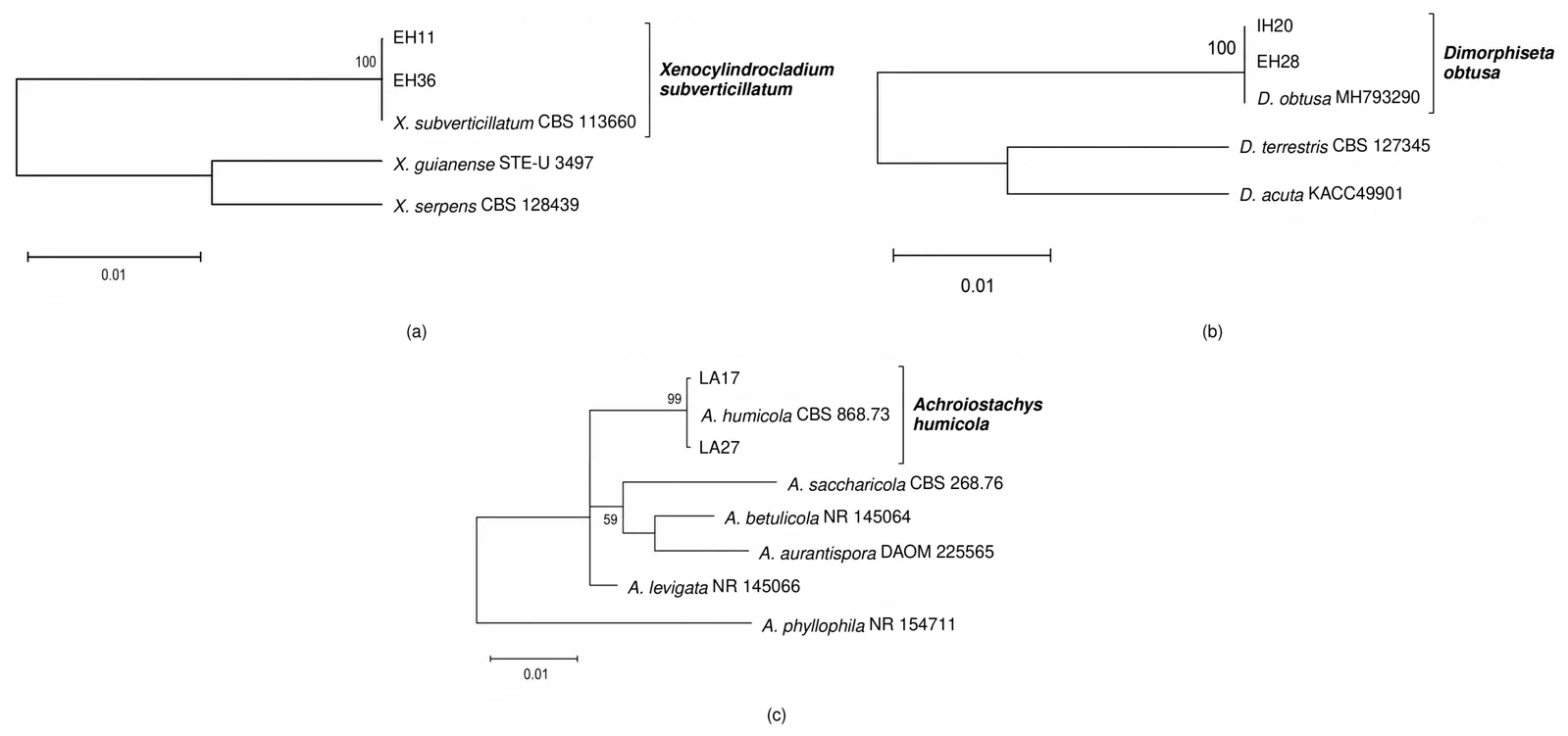
Maximum Likelihood tree inferred from ITS sequences of *Xenocylindrocladium subverticillatum* (**a**), *Dimorphiseta obtusa* (**b**), and *Achroiostachys humicola* (**c**) isolated from leaves of *A. compressus*. Bootstrap values greater than or equal to 50% are indicated.

**Figure 8:**
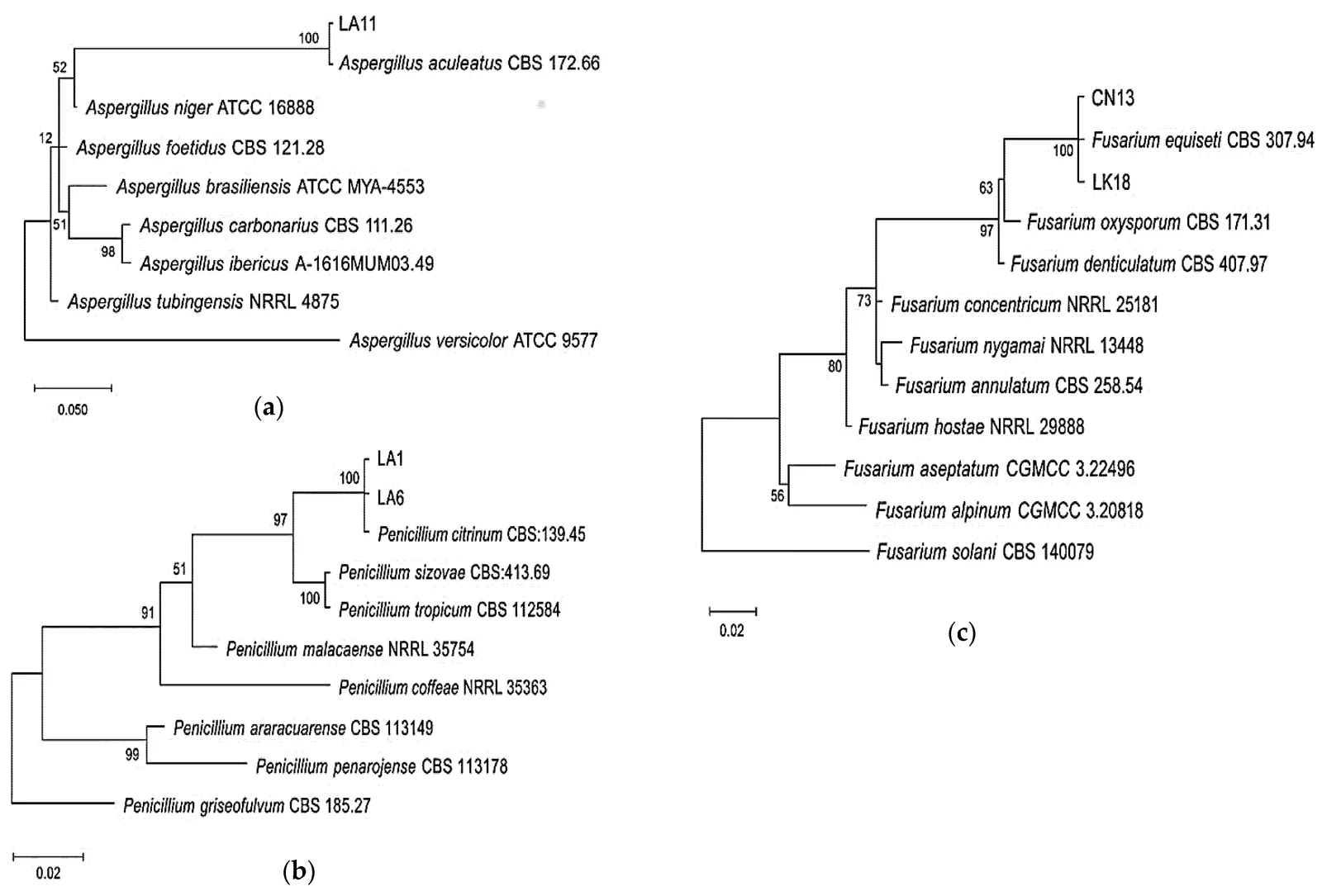
Maximum Likelihood tree inferred from ITS sequences of *Aspergillus aculeatus* (**a**), *Penicillium citrinum* (**b**), and *Fusarium equiseti* (**c**) isolated from leaves of *A. compressus*. Bootstrap values greater than or equal to 50% are indicated.

**Figure 9:**
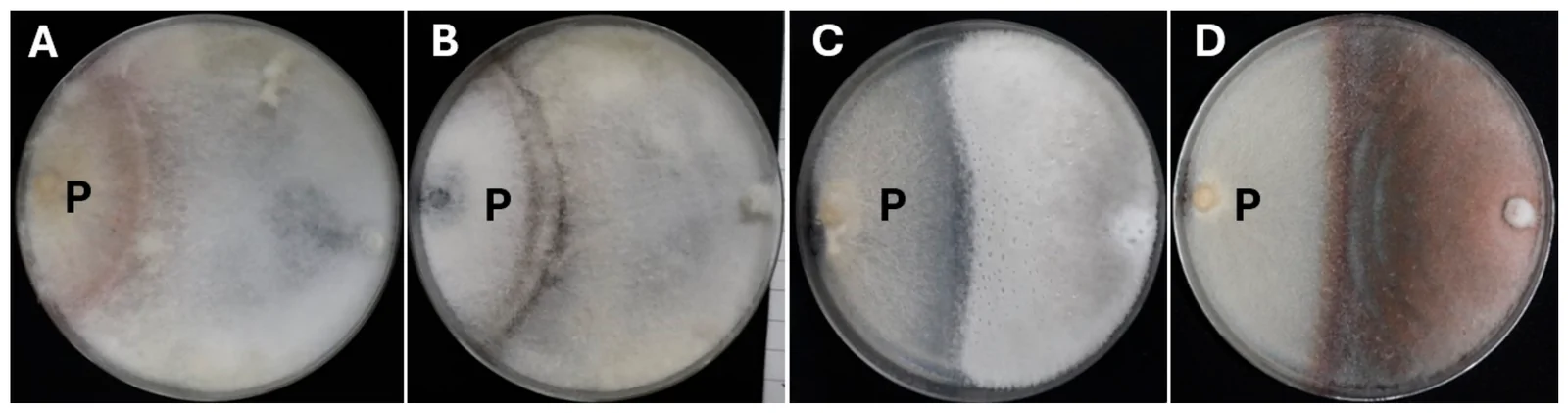
Antagonistic activity shown by some endophytic fungi isolated from *A. compressus* against banana crown rot pathogens with moderate interactions. Endophytic *D. shawiae* LA15 inhibited pathogenic *C. siamense* KL89 (**A**) and pathogenic *F. verticilliodes* NG52 (**B**); Endophytic *C. gloeosporioides* LK11 inhibited pathogenic *F. verticilliodes* NG52 (**C**); Endophytic *B. oryzae* LA21 inhibited pathogenic *F. verticilliodes* NG52 (**D**). P: Pathogenic fungi.

**Table 1:** Molecular identification of endophytic fungi isolated from leaves of *A. compressus*.

No.	Isolate	Morphological Identification	GenBank Accession Number					Similarity (%)	Query Coverage (%)	Species Identity
			ITS	GAPDH	β-Tubulin	TEF-1α	ACT			
1	LK1	*Curvularia* sp.	PQ580754	PX111929	-	-	-	100	ITS, GAPDH: 100	*C. lunata*
2	LK2	*Curvularia* sp.	PQ580757	PX111923	-	-	-	99	ITS, GAPDH: 100	*C. lunata*
3	LK14	*Curvularia* sp.	PQ580767	PX111922	-	-	-	99	ITS, GAPDH: 100	*C. lunata*
4	LK17	*Curvularia* sp.	PQ580758	PX111924	-	-	-	100	ITS, GAPDH: 100	*C. lunata*
5	LK23	*Curvularia* sp.	PQ580756	PX111921	-	-	-	100	ITS, GAPDH: 100	*C. lunata*
6	CN2	*Curvularia* sp.	PQ580764	PX111927	-	-	-	99	ITS, GAPDH: 100	*C. lunata*
7	CN14	*Curvularia* sp.	PQ580763	PX111928	-	-	-	100	ITS, GAPDH: 100	*C. lunata*
8	IH3	*Curvularia* sp.	PQ580768	PX111917	-	-	-	99	ITS, GAPDH: 100	*C. lunata*
9	IH13	*Curvularia* sp.	PQ580759	PX111916	-	-	-	100	ITS, GAPDH: 100	*C. lunata*
10	IH21	*Curvularia* sp.	PQ580760	PX111915	-	-	-	99	ITS, GAPDH: 100	*C. lunata*
11	Li8	*Curvularia* sp.	PQ580765	PX111920	-	-	-	100	ITS, GAPDH: 100	*C. lunata*
12	LA3	*Curvularia* sp.	PQ580766	PX111919	-	-	-	99	ITS, GAPDH: 100	*C. lunata*
13	LA7	*Curvularia* sp.	PQ580762	PX111925	-	-	-	100	ITS, GAPDH: 100	*C. lunata*
14	LA29	*Curvularia* sp.	PQ580761	PX111926	-	-	-	100	ITS, GAPDH: 100	*C. lunata*
15	EH21	*Curvularia* sp.	PQ580755	PX111918	-	-	-	99	ITS, GAPDH: 100	*C. lunata*
16	EH18	*Curvularia* sp.	PQ580772	PQ809532	-	-	-	99	ITS, GAPDH: 100	*C.geniculata*
17	EH2	*Curvularia* sp.	PQ580769	PQ809530	-	-	-	99	ITS, GAPDH: 100	*C. geniculata*
18	EH30	*Curvularia* sp.	PQ580770	PQ809531	-	-	-	99	ITS, GAPDH: 100	*C. geniculata*
19	LA4	*Curvularia* sp.	PQ580771	PQ809533	-	-	-	99	ITS, GAPDH: 100	*C. geniculata*
20	LA5	*Curvularia* sp.	PQ580818	PX111914	-	-	-	99	ITS, GAPDH: 100	*C. akaiiensis*
21	EH17	*Curvularia* sp.	PQ580801	PX111930	-	-	-	99	ITS, GAPDH: 100	*C. ischaemi*
22	Li4	*Colletotrichum* sp.	PQ581165	PQ809527	-	-	-	99	ITS, GAPDH: 100	*C. gloeosporioides*
23	CN4	*Colletotrichum* sp.	PQ581166	PQ809526	-	-	-	99	ITS, GAPDH: 100	*C. gloeosporioides*
24	LK11	*Colletotrichum* sp.	PQ581037	PQ809525	-	-	-	99	ITS, GAPDH: 100	*C. gloeosporioides*
25	LK16	*Colletotrichum* sp.	PQ581038	PQ809524	-	-	-	99	ITS, GAPDH: 100	*C. gloeosporioides*
26	LK12	*Colletotrichum* sp.	PQ581031	PQ824062	-	-	-	99	ITS: 85.69; GAPDH: 100	*C. siamense*
27	LK22	*Colletotrichum* sp.	PQ581039	PQ824060	-	-	-	99	ITS: 85.69; GAPDH: 100	*C. siamense*
28	LK20	*Colletotrichum* sp.	PQ581167	PQ824061	-	-	-	99	ITS, GAPDH: 100	*C. siamense*
29	CN15	*Colletotrichum* sp.	PQ581034	PQ824063	-	-	-	100	ITS: 85.69; GAPDH: 100	*C. siamense*
30	EH33	*Colletotrichum* sp.	PQ581032	PX206230	-	-	-	99	ITS: 85.69; GAPDH: 100	*C. siamense*
31	CN1	*Colletotrichum* sp.	PQ581035	PQ824052	-	-	-	100	ITS: 90.24; GAPDH: 100	*C. grevilleae*
32	Li16	*Colletotrichum* sp.	PQ581168	PQ824053				100	ITS: 94.50; GAPDH: 100	*C. grevilleae*
33	LK21	*Colletotrichum* sp.	PQ581036	PQ809528	-	-	-	99	ITS, GADPH: 100	*C. queenslandicum*
34	EH20	*Colletotrichum* sp.	PQ581033	PQ809529	-	-	-	99	ITS, GADPH: 100	*C. queenslandicum*
35	EH26	*Colletotrichum* sp.	PQ580819	PX111913	-	-	-	99	ITS, GADPH: 100	*C. gigasporum*
36	LK18	*Fusarium* sp.	PQ476210	-	-	-	-	99	ITS: 100	*F. equiseti*
37	CN13	*Fusarium* sp.	PQ476209	-	-	-	-	99	ITS:100	*F. equiseti*
38	LA1	*Penicillium* sp.	PQ475970	-	-	-	-	99	ITS:100	*P. citrinum*
39	LA6	*Penicillium* sp.	PQ475971	-	-	-	-	99	ITS:100	*P. citrinum*
40	LA11	*Aspergillus* sp.	PQ475943	-	-	-	-	99	ITS:100	*A. aculeatus*
41	EH22	*Xylaria* sp.	PQ476016	-	-	-	PQ820480	99	ITS: 100; ACT: 99.66	*X. feejeensis*
42	LA12	Morphotype 1	PQ475998	-	PX111912	PX111895	-	99	ITS: 100; β-tubulin: 94.93; TEF-1α: 100	*Diaporthe shawiae*
43	LA13	Morphotype 1	PQ475997	-	PX111910	PX111897	-	99	ITS: 100; β-tubulin: 94.93; TEF-1α: 100	*D. shawiae*
44	LA14	Morphotype 1	PQ475996	-	PX111909	PX111898	-	99	ITS: 100; β-tubulin: 94.93; TEF-1α: 100	*D. shawiae*
45	LA15	Morphotype 1	PQ475995	-	PX111908	PX111899	-	99	ITS: 100; β-tubulin: 94.93; TEF-1α: 100	*D. shawiae*
46	LA16	Morphotype 1	PQ475994	-	PX111907	PX111900	-	99	ITS: 100; β-tubulin: 94.93; TEF-1α: 100	*D. shawiae*
47	LA24	Morphotype 1	PQ475993	-	PX111906	PX111901	-	99	ITS: 100; β-tubulin: 94.93; TEF-1α: 100	*D. shawiae*
48	LA25	Morphotype 1	PQ475990	-	PX111905	PX111902	-	99	ITS: 100; β-tubulin: 94.93; TEF-1α: 100	*D. shawiae*
49	LA26	Morphotype 1	PQ475992	-	PX111904	PX111903	-	99	ITS: 100; β-tubulin: 94.93; TEF-1α: 100	*D. shawiae*
50	LA28	Morphotype 1	PQ475991	-	PX111911	PX111896	-	99	ITS: 100; β-tubulin: 94.93; TEF-1α: 100	*D. shawiae*
51	EH34	Morphotype 2	PQ475978	-	-	-	-	99	ITS: 100	*Stagonospora* *bicolor*
52	EH7	Morphotype 2	PQ475979	-	-	-	-	99	ITS: 100	*S. bicolor*
53	EH12	Morphotype 2	PQ475980	-	-	-	-	99	ITS: 100	*S. bicolor*
54	EH31	Morphotype 2	PQ475981	-	-	-	-	99	ITS: 100	*S. bicolor*
55	CN16	Morphotype 2	PQ475982	-	-	-	-	99	ITS: 100	*S. bicolor*
56	CN9	Morphotype 2	PQ475983	-	-	-	-	99	ITS: 100	*S. bicolor*
57	CN19	Morphotype 2	PQ475984	-	-	-	-	99	ITS: 100	*S. bicolor*
58	Li12	Morphotype 2	PQ475985	-	-	-	-	99	ITS: 100	*S. bicolor*
59	Li10	Morphotype 2	PQ475986	-	-	-	-	99	ITS: 100	*S. bicolor*
60	Li3	Morphotype 2	PQ475987	-	-	-	-	99	ITS: 100	*S. bicolor*
61	Li2	Morphotype 2	PQ475977	-	-	-	-	99	ITS: 100	*S. bicolor*
62	LK8	Morphotype 2	PQ475975	-	-	-	-	99	ITS: 100	*S. bicolor*
63	LK6	Morphotype 2	PQ475976	-	-	-	-	99	ITS: 100	*S. bicolor*
64	LK10	Morphotype 3	PQ475956	-	-	-	-	99	ITS:90.57	*Bipolaris oryzae*
65	LA9	Morphotype 3	PQ475957	-	-	-	-	99	ITS:90.57	*B. oryzae*
66	LA21	Morphotype 3	PQ475958	-	-	-	-	99	ITS:90.57	*B. oryzae*
67	IH16	Morphotype 3	PQ475959	-	-	-	-	99	ITS:90.57	*B. oryzae*
68	EH8	Morphotype 4	PQ475967	-	-	-	-	99	ITS:100	*Muyocopron* *laterale*
69	EH16	Morphotype 4	PQ475966	-	-	-	-	99	ITS:100	*M. laterale*
70	EH25	Morphotype 4	PQ475968	-	-	-	-	99	ITS:100	*M. laterale*
71	Li17	Morphotype 4	PQ475965	-	-	-	-	99	ITS:100	*M. laterale*
72	LA27	Morphotype 5	PQ475933	-	-	-	-	99	ITS:100	*Achroiostachys humicola*
73	LA17	Morphotype 5	PQ475934	-	-	-	-	99	ITS:100	*A. humicola*
74	EH35	Morphotype 6	PQ475953	-	-	-	-	99	ITS:100	*Beltraniella* *endiandrae*
75	EH27	Morphotype 6	PQ475952	-	-	-	-	99	ITS:100	*B. endiandrae*
76	IH20	Morphotype 7	PQ475962	-	-	-	-	99	ITS:100	*Dimorphiseta* *obtusa*
77	EH28	Morphotype 7	PQ475963	-	-	-	-	99	ITS:100	*D. obtusa*
78	EH11	Morphotype 8	PQ476264	-	-	-	-	99	ITS:100	*Xenocylindrocladium subverticillatum*
79	EH36	Morphotype 8	PQ476265	-	-	-	-	99	ITS:100	*X. subverticillatum*

**Table 2:** Antagonistic activity of endophytic fungi against banana crown rot pathogen in dual-culture test with PGI and types of interactions.

Endophytic Fungi	Banana Crown Rot Pathogen							
	PGI (%) and Types of Interaction							
	*F. proliferatum*		*F. verticilliodes*		*N. ipomoeae*		*C. siamense*	
*C. akaiiensis* LA5	33.89 ± 0.96 ^d^	A	56.66 ± 0.83 ^g^	A	55.89 ± 0.89 ^e^	B	55.05 ± 0.87 ^i^	A
*C. lunata* IH3	49.44 ± 0.96 ^f^	E	52.38 ± 0.83 ^f^	D	68.20 ± 0.89 ^i^	B	62.63 ± 0.88 ^j^	B
*C. geniculata* EH18	50.56 ± 0.96 ^f^	E	57.14 ± 0.00 ^gh^	D	61.03 ± 0.89 ^f^	D	55.56 ± 0.87 ^i^	B
*C.gloeosporioides* LK11	57.78 ± 0.96 ^g^	E	59.52 ± 0.83 ^h^	D	62.57 ± 0.89 ^fg^	E	55.56 ± 0.87 ^i^	D
*C. siamense* CN15	50.56 ± 0.96 ^f^	E	50.48 ± 0.83 ^ef^	D	64.11 ± 0.89 ^gh^	E	49.49 ± 0.88 ^gh^	D
*F. equiseti* CN13	58.89 ± 0.96 ^g^	B	59.52 ± 0.83 ^h^	D	65.13 ± 0.88 ^gh^	B	57.07 ± 0.88 ^i^	D
*X. feejeensis* EH22	44.44 ± 0.96 ^e^	E	35.24 ± 0.82 ^c^	B	29.74 ± 1.77 ^b^	C	39.90 ± 0.88 ^e^	A
*P. citrinum* LA1	27.78 ± 0.96 ^c^	E	30.00 ± 1.43 ^b^	E	29.74 ± 0.89 ^b^	D	39.90 ± 0.88 ^e^	D
*D. shawiae* LA15	50.56 ± 0.96 ^f^	B	70.00 ± 1.43 ^i^	B	64.11 ± 0.89 ^gh^	B	66.16 ± 0.88 ^k^	B
*S. bicolor* EH34	33.89 ± 0.96 ^d^	D	49.52 ± 0.83 ^e^	D	41.03 ± 0.89 ^c^	D	41.41 ± 0.87 ^e^	D
*B. oryzae* LA21	50.56 ± 0.96 ^f^	D	57.62 ± 0.83 ^gh^	A	65.64 ± 0.88 ^hi^	E	46.97 ± 0.00 ^f^	A
*M. laterale* EH16	16.67 ± 0.00 ^b^	E	28.57 ± 0.00 ^b^	E	38.97 ± 0.89 ^c^	E	16.67 ± 0.00 ^c^	E
*A. humicola* LA27	42.22 ± 0.96 ^e^	E	49.52 ± 0.83 ^e^	D	45.13 ± 0.88 ^d^	D	39.90 ± 0.88 ^e^	D
*B.endiandrae* EH27	58.89 ± 0.96 ^g^	E	42.38 ± 0.83 ^d^	D	64.11 ± 0.89 ^gh^	E	47.47 ± 0.87 ^fg^	D
*D. obtusa* IH20	42.22 ± 0.96 ^e^	E	49.52 ± 0.83 ^e^	A	53.34 ± 0.89 ^e^	A	25.25 ± 0.88 ^d^	B
*X. subverticillatum* EH11	50.56 ± 0.96 ^f^	E	50.00 ± 0.00 ^ef^	D	47.18 ± 0.89 ^d^	D	50.51 ± 0.88 ^h^	D
Control	00.00 ^a^	-	00.00 ^a^	-	00.00 ^a^	-	00.00 ^a^	-

Mean of three replicates; values followed by the same letter are not significantly different (*p* < 0.05) according to Tukey’s test. Types of interactions (A) intermingling of pathogen and endophytic fungi; (B) endophytic fungi overgrow the pathogen; (C) pathogen overgrows the endophytic fungi; (D) mutual inhibition with a distinct inhibition zone at a small distance <2 mm; (E) mutual inhibition with a clear inhibition zone at a greater distance >2 mm.

**Table 3:** Extracellular enzyme activity indices of endophytic fungi isolated from *A. compressus*.

Isolate	Enzyme Activity Index				
	Cellulase	Protease	Lipase	Pectinase	Amylase
*C. ischaemi* EH17	1.13 ± 0.02 ^ef^	2.23 ± 0.57 ^ef^	0.0 ± 0.0 ^a^	1.28 ± 0.05 ^bc^	1.62 ± 0.06 ^f^
*C. akaiiensis* LA5	1.11 ± 0.01 ^cdef^	1.76 ± 0.27 ^cde^	0.0 ± 0.00 ^a^	1.27 ± 0.03 ^bc^	1.52 ± 0.03 ^e^
*C. lunata* IH3	1.12 ± 0.00 ^cdef^	2.36 ± 0.24 ^ef^	0.0 ± 0.00 ^a^	1.18 ± 0.01 ^b^	1.31 ± 0.03 ^cd^
*C. geniculata* EH18	1.11 ± 0.01 ^cdef^	2.12 ± 0.23 ^def^	0.0 ± 0.00 ^a^	1.17 ± 0.01 ^b^	1.32 ± 0.02 ^d^
*C. gloeosporioides* LK11	1.09 ± 0.02 ^cde^	1.11 ± 0.03 ^bc^	1.59 ± 0.02 ^ef^	1.16 ± 0.02 ^b^	0.0 ± 0.00 ^a^
*C. siamense* CN15	1.08 ± 0.00 ^cd^	1.32 ± 0.22 ^bc^	1.61 ± 0.13 ^f^	1.16 ± 0.01 ^b^	0.0 ± 0.00 ^a^
*F. equiseti* CN13	0.75 ± 0.00 ^b^	1.34 ± 0.13 ^bc^	1.50 ± 0.06 ^def^	0.0 ± 0.00 ^a^	0.0 ± 0.00 ^a^
*X. feejeensis* EH22	1.32 ± 0.02 ^g^	1.75 ± 0.08 ^cde^	1.33 ± 0.06 ^cd^	1.22 ± 0.09 ^bc^	1.15 ± 0.01 ^b^
*P. citrinum* LA1	0.00 ± 0.00 ^a^	1.38 ± 0.06 ^bc^	1.18 ± 0.03 ^bc^	1.23 ± 0.03 ^bc^	0.0 ± 0.00 ^a^
*D. shawiae* LA15	1.12 ± 0.01 ^def^	1.28 ± 0.07 ^bc^	0.0 ± 0.00 ^a^	0.0 ± 0.00 ^a^	0.0 ± 0.00 ^a^
*S. bicolor* EH34	0.0 ± 0.00 ^a^	1.30 ± 0.00 ^bc^	1.23 ± 0.03 ^bc^	0.0 ± 0.00 ^a^	1.17 ± 0.03 ^b^
*B. oryzae* LA21	1.07 ± 0.00 ^c^	2.46 ± 0.24 ^f^	0.00 ± 0.00 ^a^	1.33 ± 0.12 ^c^	1.33 ± 0.12 ^d^
*M. laterale* EH16	1.30 ± 0.00 ^g^	2.10 ± 0.37 ^def^	1.42 ± 0.12 ^de^	1.87 ± 0.14 ^d^	0.0 ± 0.00 ^a^
*A. humicola* LA27	1.16 ± 0.01 ^f^	1.47 ± 0.21 ^bcd^	1.14 ± 0.02 ^b^	1.15 ± 0.04 ^b^	0.0 ± 0.00 ^a^
*B. endiandrae* EH27	1.08 ± 0.00 ^cde^	1.25 ± 0.05 ^bc^	1.11 ± 0.02 ^b^	0.0 ± 0.00 ^a^	1.22 ± 0.03 ^bc^
*D. obtusa* IH20	1.07 ± 0.00 ^c^	1.06 ± 0.02 ^b^	0.0 ± 0.00 ^a^	1.29 ± 0.04 ^bc^	1.15 ± 0.02 ^b^
*X. subverticillatum* EH11	0.0 ± 0.00 ^a^	1.47 ± 0.32 ^bcd^	1.22 ± 0.03 ^bc^	0.0 ± 0.00 ^a^	0.0 ± 0.00 ^a^
Control	0.0 ± 0.00 ^a^	0.0 ± 0.00 ^a^	0.0 ± 0.00 ^a^	0.0 ± 0.00 ^a^	0.0 ± 0.00 ^a^

Means of EAI ± SD (*n* = 3) followed by the same letter are not significantly different (*p* < 0.05).

**Table 4:** Classification of extracellular enzymatic reaction category based on EAI.

EAI	Reaction Category
Higher than or equal to 2 (x ≥ 2)	Strong reaction
Less than 2, but more than 1 (1 < x < 2)	Medium reaction
Equal to or less than 1 (x ≤ 1)	Weak reaction
No zone observed (0)	No formation of a clear zone

## Data Availability

The sequence data of this study have been deposited in GenBank, and the accession numbers are presented in Table 1.

## Supplementary Materials

The following supporting information can be downloaded at https://www.mdpi.com/article/10.3390/plants15203229/s1, Table S1. Distribution of culturable endophytic fungal isolates recovered from *Axonopus compressus* leaves across six sampling locations; Table S2. References/manuals used for morphological identification; Table S3. Markers used for PCR amplification of each genus and morphotype of endophytic fungi isolated from leaves of *A. compressus*; Table S4. List of markers and primer sequences used in PCR amplifications of endophytic fungal isolates from leaves of *A. compressus*; Table S5. Best-fit nucleotide-substitution models for combined sequences used in Maximum Likelihood phylogenetic tree analysis; Table S6. Epitype strains used in the phylogenetic analysis of endophytic fungi isolated from leaves of *A. compressus.* Figure S1. Morphological characteristics of endophytic fungi isolated from *Axonopus compressus* leaves.

## Abbreviations

The following abbreviations are used in this manuscript:

USM	Universiti Sains Malaysia
PDA	Potato Dextrose Agar
PDB	Potato Dextrose Broth
ITS	Internal Transcribed Spacer regions
GAPDH	Glyceraldehyde-3-Phosphate Dehydrogenase
TUB	β-Tubulin
ACT	Actin
TEF-1α	Translation Elongation Factor 1α
PCR	Polymerase Chain Reaction
BLAST	Basic Local Alignment Search Tool
ML	Maximum Likelihood
EAI	Enzyme Activity Index
