# Supplementary material for: Revisiting the Endophytic Fungi Residing Within Axonopus compressus: Molecular Identification, Antagonistic Potential, and Extracellular Enzyme Activities
Source: Plants (Basel). 2026 Oct 21;15(20):3229. doi: 10.3390/plants15203229 (PMC13708824; doi:10.3390/plants15203229)
Supplement: Supplementary file 1 [file plants-15-03229-s001.zip › plants-4609385-supplementary.pdf]

Supplementary Figure S1. Morphological characteristics of endophytic fungi isolated from *Axonopus compressus* leaves.

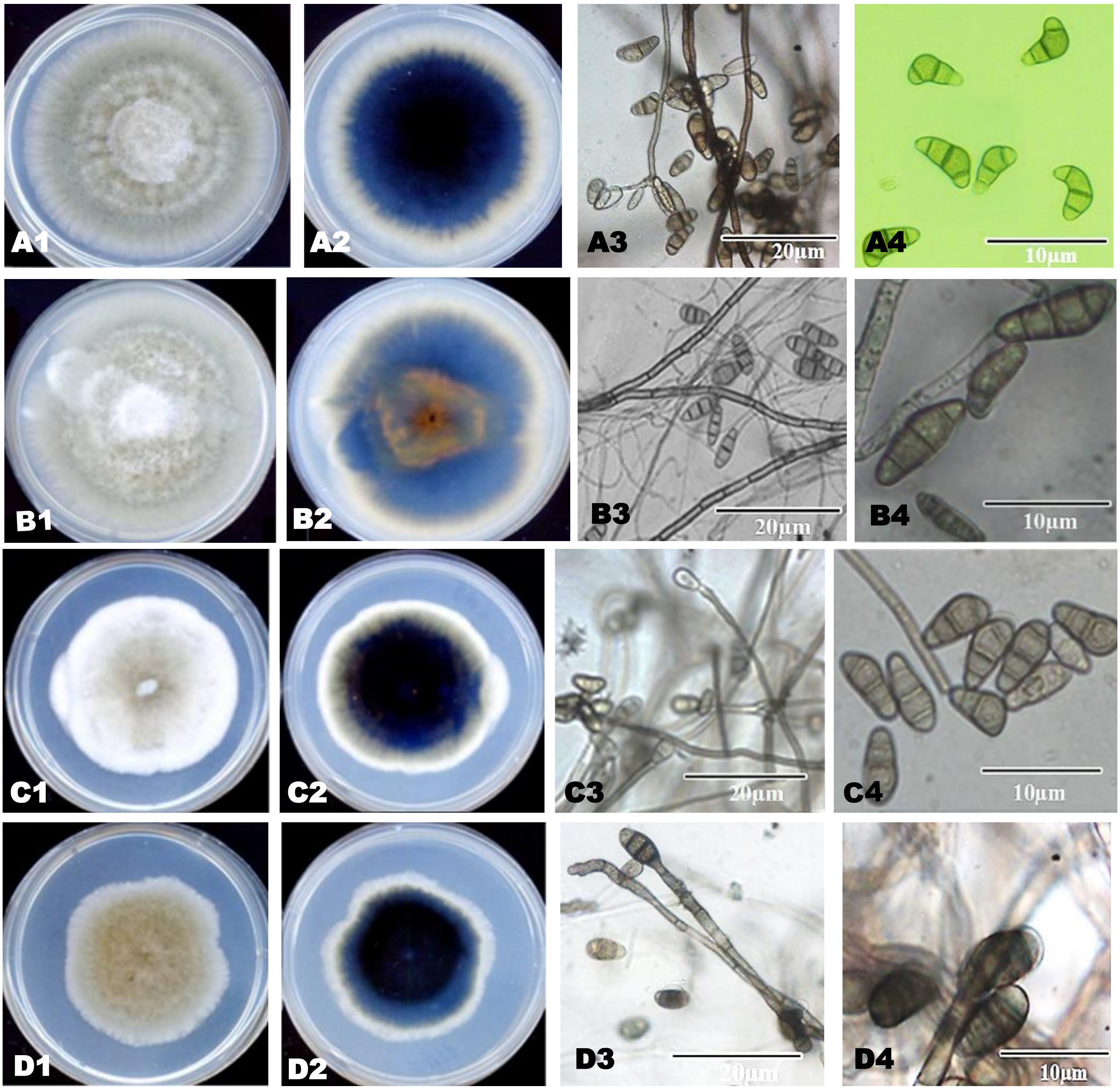

Supplemetary Figure S1a. Morphological characteristics of *Curvularia* spp. isolated from *Axonopus compressus* leaves. **(A1 – A4) *Curvularia* sp. 1 (A1)** Colony upper surface on PDA; (**A2**) Colony reverse surface on PDA; **(A3)** Conidiophore; **(A4)** Conidia; **(B1 – B4) *Curvularia* sp. 1 (B1)** Colony upper surface on PDA; **(B2)** Colony reverse surface on PDA; **(B3)** Conidiophore; **(B4)** Conidia; **(C1 – C4) *Curvularia* sp. 3 (C1)** Colony upper surface on PDA **(C2)** Colony reverse surface on PDA; **(C3)** Conidiophore; **(C4)** Conidia; **(D1 – D4) *Curvularia* sp. 4 (D1)** Colony upper surface on PDA; **(D2)** Colony reverse surface on PDA; **(D3)** Conidiophore;

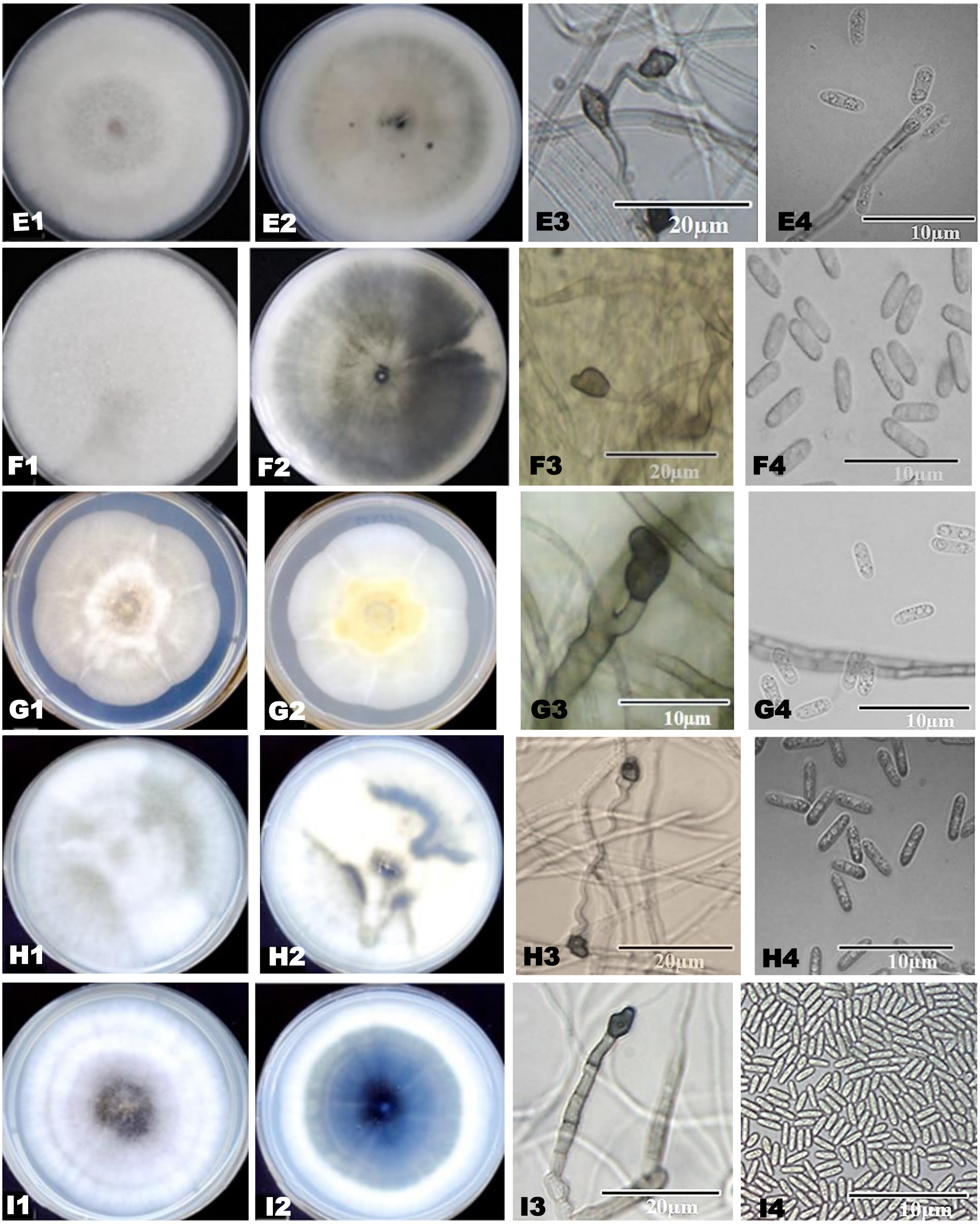

Supplementary Figure S1b. Morphological characteristics of *Colletotrichum* spp. isolated from *Axonopus compressus* leaves. **(E1–E4) *Colletotrichum* sp. 1. (E1)** Colony upper surface on PDA; **(E2)** Colony reverse surface on PDA; **(E3)** Appresoria; **(E4)** Conidia; **(F1 – F4) *Colletotrichum* sp. 2 (F1)** Colony upper surface on PDA; **(F2)** Colony reverse surface on PDA; **(F3)** Conidiophore; **(F4)** Conidia; **(G1 – G4) *Colletotrichum* sp. 3 (G1)** Colony upper surface on PDA **(G2)** Colony reverse surface on PDA; **(G3)** Appresoria; **(G4)** Conidia; **(H1 – H4) *Colletotrichum* sp 4. (H1)** Colony upper surface on PDA; **(H2)** Colony reverse surface on PDA; **(H3)** Appresoria; **(H4)** Conidia **(I1 – I4) *Colletotrichum* sp. 5 (I1)** Colony upper surface on PDA; **(I2)** Colony reverse surface on PDA; **(I3)** Appresoria; **(I4)** Conidia

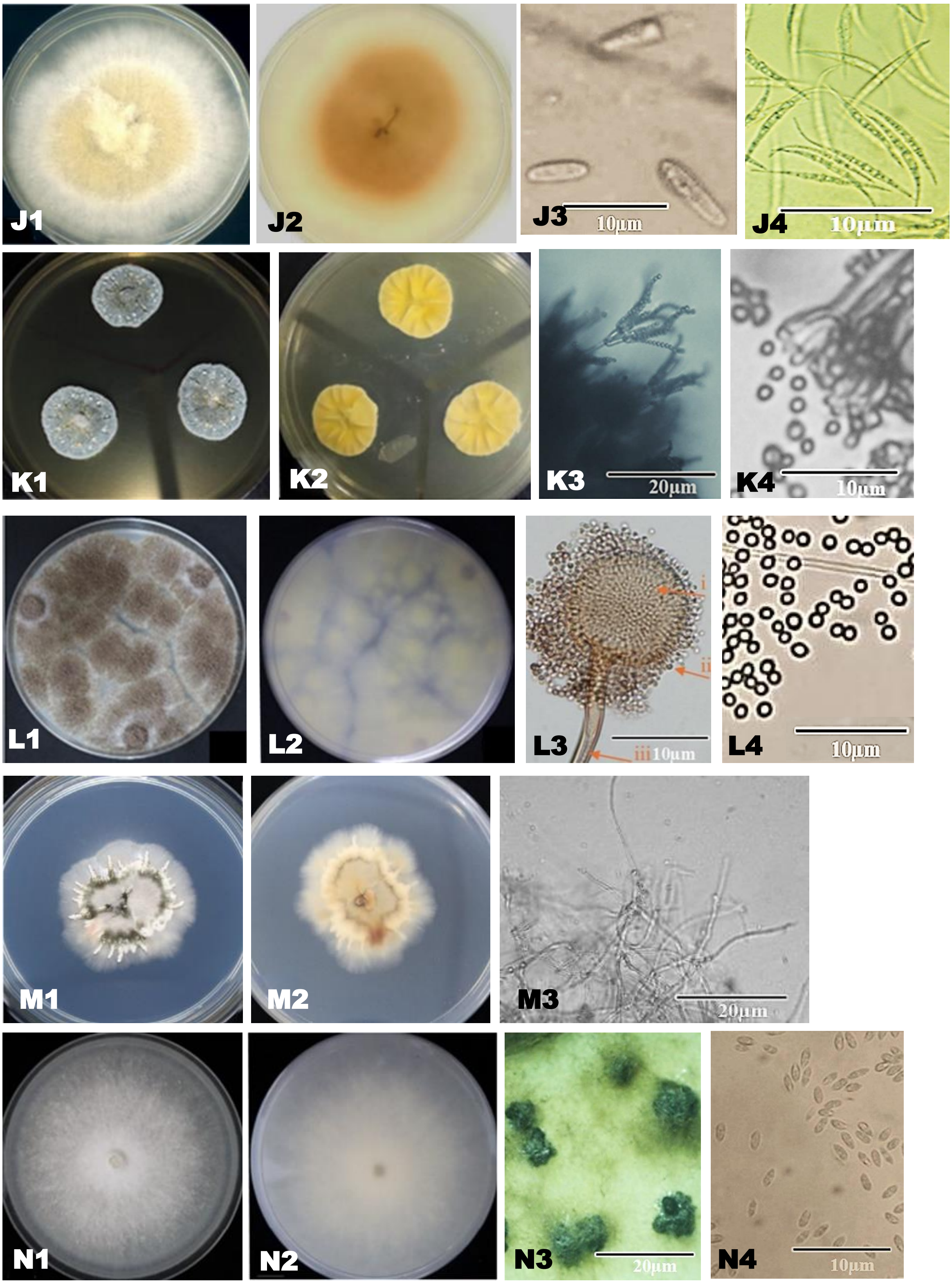

Supplementary Figure S1c. Morphological characteristics of fungi isolated from *Axonopus compressus* leaves. **(J1 – J4) *Fusarium* sp. (J1)** Colony upper surface on PDA; **(J2)** Colony reverse surface on PDA; **(J3)** Appresoria; **(J4)** Conidia; **(K1 – K4) *Penicillium* sp. (K1)** Colony upper surface on PDA; **(K2)** Colony reverse surface on PDA; **(K3)** Microconidia; **(K4)** Macroconidia; **(L1 – L4) *Aspergillus* sp. (L1)** Colony upper surface on PDA **(L2)** Colony reverse surface on PDA; **(L3)** Appresoria; **(L4)** Conidia; **(M1 – M4) *Xylaria* sp. (M1)** Colony upper surface on PDA; **(M2)** Colony reverse surface on PDA; **(M3)** Mycelia; **(N1 – N4) Morphotype 1 (N1)** Colony upper surface on PDA; **(N2)** Colony reverse surface on PDA; **(N3)** Conidiomata; **(N4)** Conidia

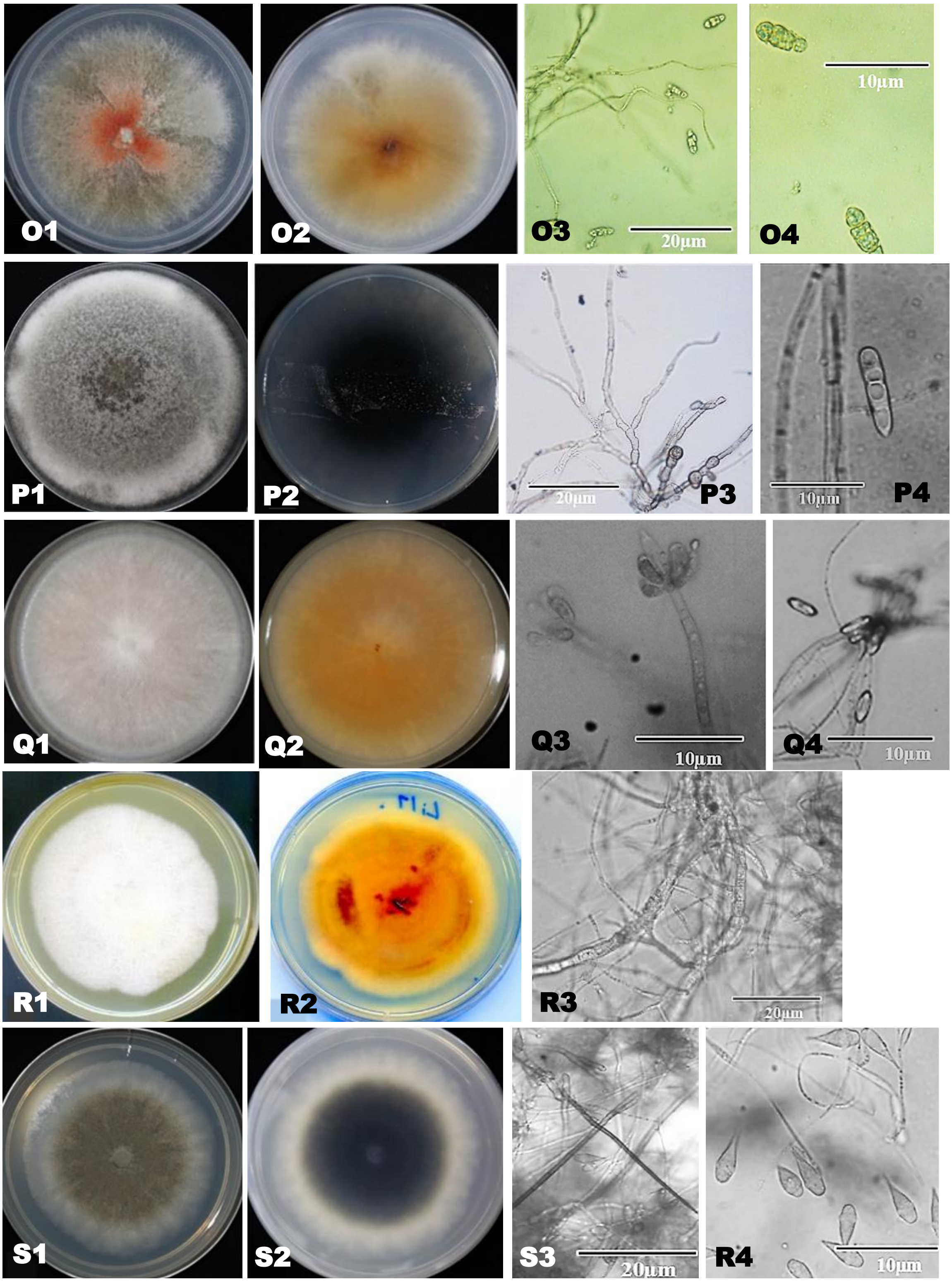

Supplementary Figure S1d. Morphological characteristics of fungi isolated from *Axonopus compressus* leaves. **(O1 – O4) Morphotype 2 (O1)** Colony upper surface on PDA; **(O2)** Colony reverse surface on PDA; **(O3)** Conidiophore; **(O4)** Conidia; **(P1 – P4) Morphotype 3 (P1)** Colony upper surface on PDA; **(P2)** Colony reverse surface on PDA; **(P3)** Conidiophore; **(P4)** Conidia; **(Q1 – Q4) Morphotype 5 (Q1)** Colony upper surface on PDA **(Q2)** Colony reverse surface on PDA; **(Q3)** Conidiophore; **(Q4)** Conidia; **(R1 – R4) Morphotype 4 (R1)** Colony upper surface on PDA; **(R2)** Colony reverse surface on PDA; **(R3)** Mycelia; **(S1 – S4) Morphotype 6 (S1)** Colony upper surface on PDA; **(S2)** Colony reverse surface on PDA; **(S3)** Conidiophore; **(S4)** Conidia

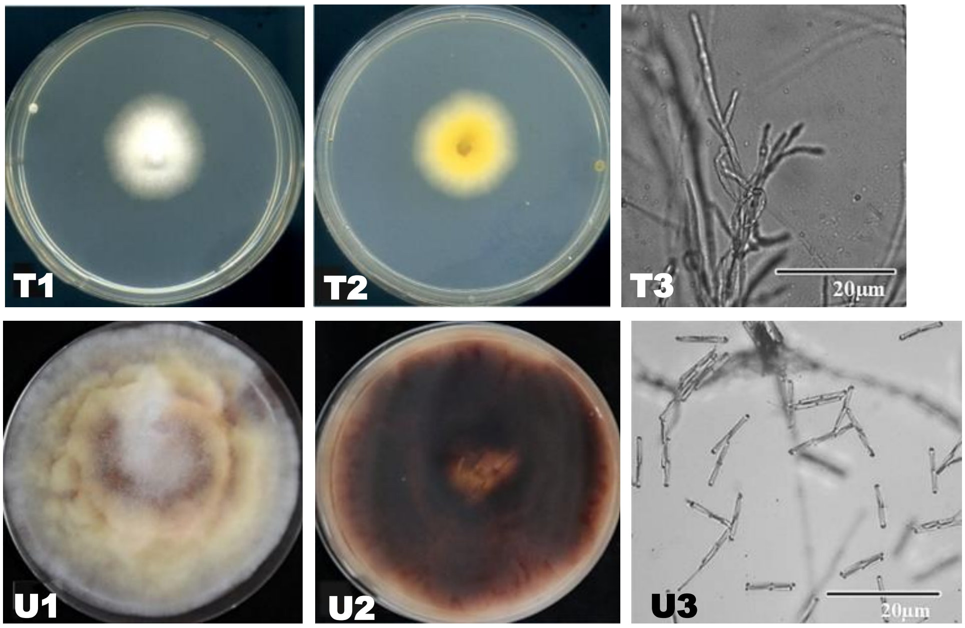

Supplementary Figure S1e. Morphological characteristics of fungi isolated from *Axonopus compressus* leaves. **(T1 – T4) Morphotype 7 (T1)** Colony upper surface on PDA; **(T2)** Colony reverse surface on PDA; **(T3)** Mycelia; **(U1 – U4) Morphotype 8 (U1)** Colony upper surface on PDA; **(U2)** Colony reverse surface on PDA; **(U3)** Conidia.

Supplementary Table S1. Distribution of culturable endophytic fungal isolates recovered from *Axonopus compressus* leaves across six sampling locations

Species	Location	Number of isolates
C. lunata	Harapan Lake (LK)	5
C. gloeosporioides	Harapan Lake (LK)	2
C. siamense	Harapan Lake (LK)	3
F. equiseti	Harapan Lake (LK)	1
C. queenslandicum	Harapan Lake (LK)	1
S. bicolor	Harapan Lake (LK)	2
B. oryzae	Harapan Lake (LK)	1
B. oryzae	VCRU laboratory lawn (LA)	2
C. lunata	VCRU laboratory lawn (LA)	3
C. geniculata	VCRU laboratory lawn (LA)	1
C. akaiiensis	VCRU laboratory lawn (LA)	1
P. citrinum	VCRU laboratory lawn (LA)	2
A. aculeatus	VCRU laboratory lawn (LA)	1
D. shawiae	VCRU laboratory lawn (LA)	9
A. humicola	VCRU laboratory lawn (LA)	2
C. lunata	Convex@USM lawn (CN)	2
C. gloeosporioides	Convex@USM lawn (CN)	1
C. siamense	Convex@USM lawn (CN)	1
C. grevilleae	Convex@USM lawn (CN)	1
F. equiseti	Convex@USM lawn (CN)	1
S. bicolor	Convex@USM lawn (CN)	3
C. lunata	International House (IH)	3
B. oryzae	International House (IH)	1
D. obtusa	International House (IH)	1
C. lunata	Eco Hub (EH)	1
C. geniculata	Eco Hub (EH)	3
C. ischaemi	Eco Hub (EH)	1
C. siamense	Eco Hub (EH)	1
C. queenslandicum	Eco Hub (EH)	1
C. gigasporum	Eco Hub (EH)	1
X. feejeensis	Eco Hub (EH)	1
S. bicolor	Eco Hub (EH)	4
M. laterale	Eco Hub (EH)	3
B. endiandrae	Eco Hub (EH)	2
D. obtusa	Eco Hub (EH)	1
X. subverticillatum	Eco Hub (EH)	2
C. gloeosporioides	Hamzah Sendut Library area (Li)	1
C. lunata	Hamzah Sendut Library area (Li)	1

C. grevilleae	Hamzah Sendut Library area (Li)	1
S. bicolor	Hamzah Sendut Library area (Li)	4
M. laterale	Hamzah Sendut Library area (Li)	1
**Total**		**79**

Supplementary Table S2. References / manuals used for morphological identification

**Fungal genera**	**References**
Culvularia spp.	McGinnis (1980); Tan et al. (2018)
Colletotrichum spp.	Weir et al (2012)
Fusarium sp.	Leslie & Summerell (2008)
Penicillium sp.	Visagie et al. (2014)
Aspergillus sp.	Samson et al. (2010)
Xylaria sp.	Chacko and Rogers (1981)

McGinnis, M. R. (1980). *Laboratory Handbook of Medical Mycology*. Academic Press.

Tan, Y.P., Crous, P.W. and Shivas, R.G. (2018). Cryptic species of *Curvularia* in the culture collection of the Queensland Plant Pathology Herbarium. *MycoKeys* (35), pp.1 – 25.

Weir, B.S., Johnston, P.R. and Damm, U. (2012). The *Colletotrichum gloeosporioides* species complex. *Studies in Mycology*, *73*, pp.115-180.

Leslie, J.F., Summerell, B.A. and Bullock, S. (2006). *The Fusarium Laboratory Manual* (Vol. 2, No. 10). Ames, IA: Blackwell Pub.

Samson, R.A., Visagie, C.M., Houbraken, J., Hong, S.B., Hubka, V., Klaassen, C.H., Perrone, G., Seifert, K.A., Susca, A., Tanney, J.B. and Varga, J., 2014. Phylogeny, identification and nomenclature of the genus *Aspergillus*. *Studies in Mycology*, *78*(1), pp.141-173.

Visagie, C.M., Houbraken, J., Frisvad, J.C., Hong, S.B., Klaassen, C.H.W., Perrone, G., Seifert, K.A., Varga, J., Yaguchi, T. and Samson, R.A., 2014. Identification and nomenclature of the genus *Penicillium. Studies in Mycology*, *78*(1), pp.343-371.

Chacko, R.J. and Rogers, J.D. (1981). Cultural characteristics of some species of *Xylaria. Mycologia*, *73*(3), pp.415-428.

Supplementary Table S3. Markers used for PCR amplification of each genus and morphotype of endophytic fungi isolated from leaves of *A. compressus*

**Fungal genera / Morphotype**	**Region / Gene**	**References**
Curvularia	ITS, GAPDH	Manamgoda et al. (2012)
Colletotrichum	ITS, GAPDH	Weir et al. (2002), Jayawardena et al. (2016)
Fusarium	ITS	Schoch et al. (2012); Samson et al. (2014), Visagie et al. (2014)
Penicillium		
Aspergillus		
Morphotypes 2 - 8		
Morphotype 1 (*Diaporthe*)	ITS, TUB2, TEF1-α	Udayanga et al. (2012); Udayanga et al. (2014)
Xylaria	ITS, ACT	Hsieh et al. (2020)

ITS (Internal Transcribed Spacer)**;** TEF1-α (Translation Elongation Factor 1-alpha); TUB2 (β-tubulin); GAPDH (Glyceraldehyde-3-phosphate dehydrogenase); ACT (Actin).

References

Hsieh, H.M., Lin, C.R., Fang, M.J., Rogers, J.D., Fournier, J., Lechat, C. and Ju, Y.M., 2010.
Jayawardena, R.S., Hyde, K.D., Damm, U., Cai, L., Liu, M., Li, X.H., Zhang, W., Zhao, W.S. and Yan, J.Y. 2016. Notes on currently accepted species of *Colletotrichum*. *Mycosphere* 7(8), pp. 1192 – 1260.

Manamgoda, D.S., Cai, L., McKenzie, E.H., Crous, P.W., Madrid, H., Chukeatirote, E., Shivas, R.G., Phylogenetic status of *Xylaria* subgenus *Pseudoxylaria* among taxa of the subfamily Xylarioideae (Xylariaceae) and phylogeny of the taxa involved in the subfamily. *Molecular Phylogenetics and Evolution*, *54*(3), pp.957-969.

Samson, R.A., Visagie, C.M., Houbraken, J., Hong, S.B., Hubka, V., Klaassen, C.H., Perrone, G., Seifert, K.A., Susca, A., Tanney, J.B. and Varga, J., 2014. Phylogeny, identification and nomenclature of the genus *Aspergillus*. *Studies in Mycology*, *78*(1), pp.141-173.

Schoch, C.L., Seifert, K.A., Huhndorf, S., Robert, V., Spouge, J.L., Levesque, C.A., Chen, W., Fungal Barcoding Consortium, Fungal Barcoding Consortium Author List, Bolchacova, E. and Voigt, K., 2012. Nuclear ribosomal internal transcribed spacer (ITS) region as a universal DNA barcode marker for Fungi. *Proceedings of the National Academy of Sciences*, *109*(16), pp.6241-6246.

Tan, Y.P. and Hyde, K.D., 2012. A phylogenetic and taxonomic re-evaluation of the *Bipolaris-Cochliobolus-Curvularia* complex. *Fungal Diversity*, 56(1), pp.131-144.

Udayanga, D., Castlebury, L.A., Rossman, A.Y., Chukeatirote, E. and Hyde, K.D., 2014. Insights into the genus *Diaporth*e: phylogenetic species delimitation in the *D. eres* species complex. *Fungal Diversity*, *67*(1), pp.203-229.

Udayanga, D., Liu, X., Crous, P.W., McKenzie, E.H., Chukeatirote, E. and Hyde, K.D., 2012. A multi-locus phylogenetic evaluation of *Diaporthe* (*Phomopsis*). *Fungal diversity*, *56*(1), pp.157-171.

Visagie, C.M., Houbraken, J., Frisvad, J.C., Hong, S.B., Klaassen, C.H.W., Perrone, G., Seifert, K.A., Varga, J., Yaguchi, T. and Samson, R.A., 2014. Identification and nomenclature of the genus *Penicillium. Studies in Mycology*, *78*(1), pp.343-371.

Supplementary Table S4. List of markers and primer sequences used in PCR amplifications of endophytic fungal isolates from leaves of *A. compressus*

Region/Gene	Primers	Primer sequence (5-3')	Fungal genera	References
ITS	ITS1	TCCGTAGGTGAACCTGCGG	All fungal genera	Templeton et al. (1992)
	ITS4	TCCTCCGCTTATTGATATGC		
GAPDH	Gpd1	CAACGGCTTCGGTCGCATTG	*Curvularia*	Carbone & Kohn (1999)
	Gpd2	GCCAAGCAGTTGGTTGTGC	*Colletotrichum*	
ACT	ACT-512F	ATGTGCAAGGCCGGTTTCG	*Xylaria*	O'Donnell & Cigelnik (1997)
	ACT-783R	TACGAGTCCTTCTGGCCCAT		
β-tubulin	bt2a	GGTAA CAAAT GGTGCTTTC	*Diaporthe*	Carbone & Kohn (1999); O'Donnell et al. (1998)
	bt2b	ACCCTCAGTGTAGTGACCCTTGGC		
TEF-1α	EF1-728F	CATCGAGAAGTTCGAGAAGG		Templeton et al. (1992)
	EF1-986R	TACTTGAAGGAACCCTTACC		

Supplementary Table S5. Best-fit nucleotide-substitution models for combined sequences used in Maximum Likelihood phylogenetic tree analysis

**Fungal species**	**Sequences (region / gene)**	**Best-fit model**
Curvularia spp.	Combined ITS and GAPDH	Kimura 2-parameter
Colletotrichum spp.	Combined ITS and GAPDH	Kimura 2-parameter
Fusarium equiseti	ITS	Jukes–Cantor
Penicillium citrinum		
Achroiostachys humicola		
Dimorphiseta obtusa		
Xylaria feejeensis	Combined ITS and ACT	Kimura 2-parameter
Diaporthe shawiae	Combined ITS, TEF-1α and β-tubulin	Hasegawa–Kishino–Yano
Stagonospora bicolor	ITS	Kimura 2-parameter
Bipolaris. oryzae		
Beltraniella endiandrae		
Xenocylindrocladium subverticillatum		
Aspergillus aculeatus	ITS	Tamura 3-parameter
Muyocopron laterale		

Supplementary Table S6. Epitype strains used in the phylogenetic analysis of endophytic fungi isolated from leaves of *A. compressus*

Species	GenBank Accession Number				
	**ITS**	**GAPDH**	**TEF-1α**	**β-tubulin**	**ACT**
Curvularia lunata	CBS 730.96	CBS 730.96	-	-	-
Curvularia geniculata	BRIP: 72496a	BRIP: 72496a	-	-	-
Curvularia ischaemi	CBS 630.82	CBS 630.82	-	-	-
Curvularia akaiiensis	CBS 127726	CBS 127726	-	-	-
Colletotrichum siamense	MFLU 090230	MFLU 090230	-	-	-
Colletotrichum gloeosporioides	ICMP 17821	ICMP 17821	-	-	-
Colletotrichum gigasporum	CBS 133266	CBS 133266	-	-	-
Colletotrichum queenslandicum	ICMP 1778	ICMP 1778	-	-	-
Colletotrichum grevilleae	CBS 132879	CBS 132879	-	-	-
Fusarium equiseti	NRRL 26419	-	-	-	-
Penicillium citrinum	CBS:139.45	-	-	-	-
Aspergillus aculeatus	CBS 172.66	-	-	-	-
Xylaria feejeensis	MFLUCC17-0011	-	-	-	GQ449241
Diaporthe shawiae	KX013187	-	BRIP 64534a	BRIP 64534a	
Stagonospora bicolor	ATCC 42652	-	-	-	-
Bipolaris oryzae	CBS:127242	-	-	-	-
Beltraniella endiandrae	CBS 137976	-	-	-	-
Achroiostachys humicola	CBS 868.73	-	-	-	-
Muyocopron laterale	CBS_145313	-	-	-	-
Dimorphiseta obtusa	MH793290	-	-	-	-
Xenocylindrocladium subverticillatum	CBS:113660	-	-	-	-
